# UV–Vis Absorption Properties of New Aromatic Imines and Their Compositions with Poly({4,8-bis[(2-Ethylhexyl)oxy]Benzo[1,2-b:4,5-b′]Dithiophene-2,6-diyl}{3-Fluoro-2-[(2-Ethylhexyl)Carbonyl]Thieno[3,4-b]Thiophenediyl})

**DOI:** 10.3390/ma12244191

**Published:** 2019-12-13

**Authors:** Agnieszka Gonciarz, Robert Pich, Krzysztof Artur Bogdanowicz, Beata Jewloszewicz, Wojciech Przybył, Karolina Dysz, Agnieszka Dylong, Anna Kwak, Andrzej Kaim, Agnieszka Iwan, Jaroslaw Rusin, Adam Januszko

**Affiliations:** 1 General Tadeusz Kosciuszko Military University of Land Forces, Wroclaw; MULF Wroclaw, Faculty of Security and Safety Research, Czajkowskiego 109 Str., 51–147 Wroclaw, Poland; agnieszka.gonciarz@awl.edu.pl (A.G.); robert.pich@awl.edu.pl (R.P.); jaroslaw.rusin@awl.edu.pl (J.R.); 2 Military Institute of Engineer Technology, Obornicka 136 Str., 50–961 Wroclaw, Poland; bogdanowicz@witi.wroc.pl (K.A.B.); jewloszewicz@witi.wroc.pl (B.J.); przybyl@witi.wroc.pl (W.P.); dysz@witi.wroc.pl (K.D.); dylong@witi.wroc.pl (A.D.); kwak@witi.wroc.pl (A.K.); januszko@witi.wroc.pl (A.J.); 3 Faculty of Chemistry, University of Warsaw, Pasteura 1, 02–093 Warsaw, Poland; akaim@chem.uw.edu.pl

**Keywords:** imines, PTB7, UV–Vis, molar absorption coefficient, organic solar cells, thermographic camera

## Abstract

In this paper, four new aromatic imines containing at least one thiazole-based heterocycle were analyzed in detail by UV–Vis spectroscopy, taking into consideration their chemical structures and interactions with PTB7, a known polymeric electron donor widely used in bulk heterojunction organic solar cells. It is demonstrated that the absorption spectra of the investigated active compositions can be modified not only by changing the chemical structure of imine, but also via formulations with PTB7. For all investigated imines and PTB7:imine compositions, calibration curves were obtained in order to find the optimum concentration in the composition with PTB7 for expansion and optimization of absorption spectra. All imines and PTB7:imine compositions were investigated in 1,2-dichlorobenzene by UV–Vis spectroscopy in various concentrations, monitoring the changes in the π–π* and n–π* transitions. With increasing imine concentrations, we did not observe changes in absorption maxima, while with increasing imine concentrations, a hypochromic effect was observed. Finally, we could conclude that all investigated compositions exhibited wide absorptions of up to 800 nm and isosbestic points in the range of 440–540 nm, confirming changes in the macromolecular organization of the tested compounds. The theoretical calculations of their vibration spectra (FTIR) and LUMO–HOMO levels by Density Functional Theory (DFT) methods are also provided. Finally, IR thermal images were measured for organic devices based on imines and the imine:PTB7 composite.

## 1. Introduction

Almost all scientific papers dedicated to imines or poly(imine)s include UV–Vis absorption studies of investigated compounds [1,2,3,4,5,6,7,8]. In addition, complexes of Lewis or Brønsted acids with imines have been widely investigated by UV–Vis spectroscopy [1,9,10]. 

**Figure 1:**
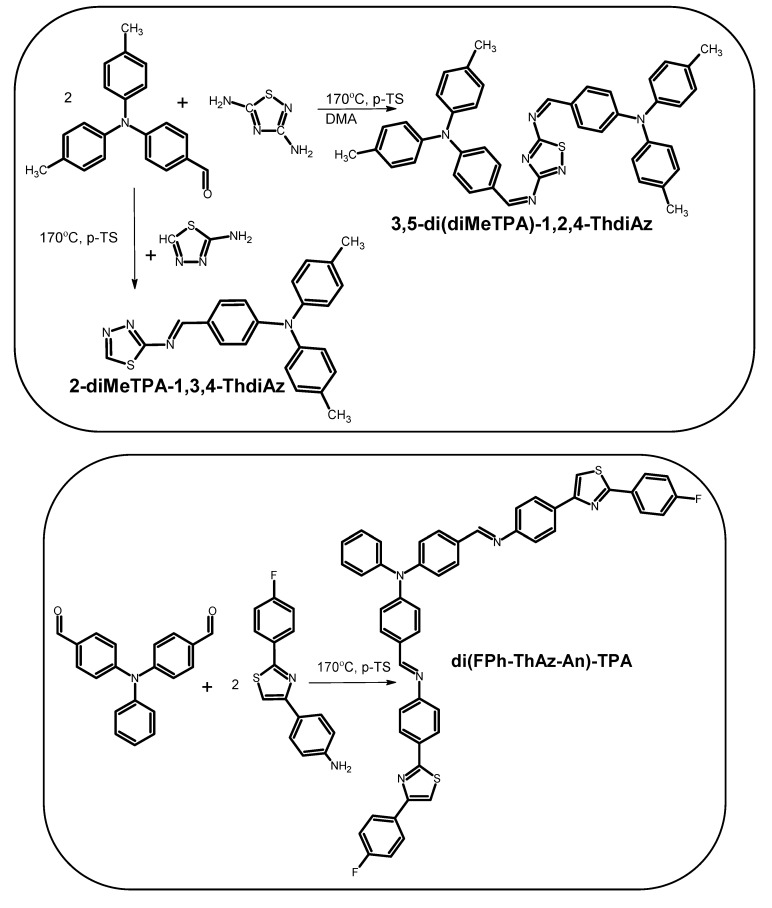

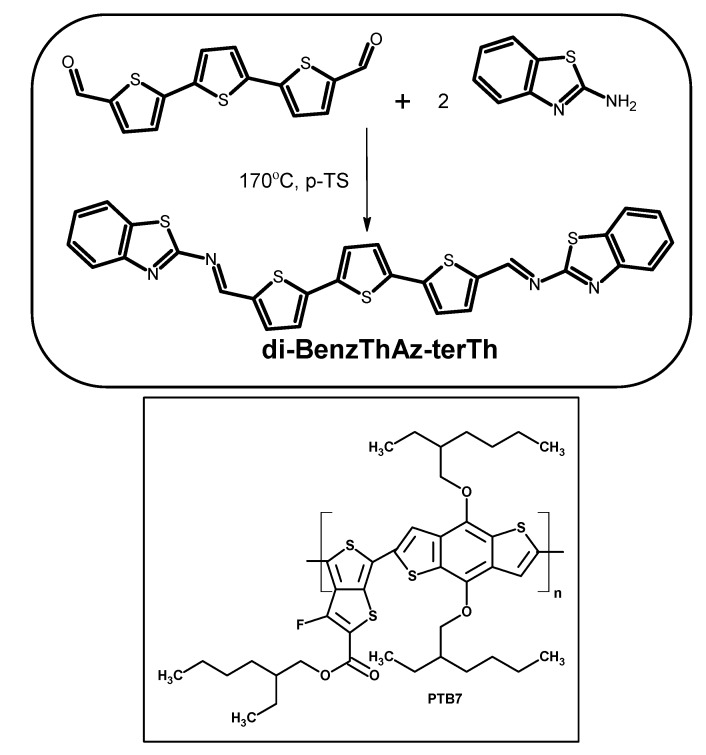
Synthetic routes of the new imines and the chemical structure of PTB7.

However, to the best of our knowledge, the deep UV–Vis of imines with poly({4,8-bis[(2-ethylhexyl)oxy]benzo[1,2-b:4,5-b′]dithiophene-2,6-diyl}{3-fluoro-2-[(2-ethylhexyl)carbonyl]thieno[3,4-b]thiophenediyl}) (PTB7) has not been investigated so far. For this reason, in our research, we focused on new symmetrical and unsymmetrical imines and PTB7 polymer applied as donor (low band-gap polymer) materials to demonstrate their potential applicability in organic polymer solar cells. The chemical structure of PTB7 presented in Figure 1 contains an electron-deficient thienothiophene unit and an electron-rich benzodithiophene unit, along with small branched side chains to improve solubility in organic solvents. PTB7 showed a strong absorption, from 550 to 750 nm, and exhibited the highest occupied molecular orbital energy level (HOMO) at −5.15 eV and the lowest unoccupied molecular orbital energy level (LUMO) at −3.31 eV [11]. Moreover, the theoretical experiments showed that in contrast with poly(3-hexylthiophene) (P3HT), the PTB7 polymer had a torsional potential almost independent of chain length due to electronic delocalization, hydrogen bonding, and the absence of side chains adjacent to inter-conjugation units [11].

As a commercially available polymer, PTB7 gives some of the highest reported efficiencies (power conversion efficiency (PCE) = 7.4% for a standard bulk-heterojunction (BHJ) polymer solar cell, PCE = 8.5% for BHJ devices with PTB7 which reached 128 kg/mol with a low polydispersity of 1.12, and PCE = 9.2% for an inverted solar cell) [12,13]. 

The future of BHJ polymer solar cells based on PTB7 points to the achieving of PCE > 10% by: (i) synthesis of PTB7 derivatives with high purity and low polydispersity, (ii) introduction of the ternary active layer based on PTB7:PC_71_BM, as well as new oligomers or polymers with donor/acceptor moieties with different absorption spectra and LUMO–HOMO energy levels between those of PTB7 and PC_71_BM [11].

It is well known that the materials in bulk heterojunction active layers in organic/polymer solar cells with matched and good donor and acceptor properties are required to obtain solar cells that work with high efficiency. At the present time, new acceptor components of active layers are very desirable for application in polymer solar cells [14,15,16,17]. A lot of work is focused on analyses of fullerene derivatives, such as of the optical, electrical, and structural properties of their active layers when they cooperate in solar cells with PTB7, taking into consideration temperature regimes or small additives [18,19,20,21]. 

Unfortunately, the photovoltaic parameters of imines are still low compared to other materials proposed as components of active layer materials. For example, the value of the power conversion efficiency (PCE) was found to be in the range 0.1–2.17% [22,23,24,25,26,27]. Petrus et al. [22] investigated organic solar cells with the architecture ITO/MoO_3_/TPA-N=CH-Th-CH=N-TPA:PC_70_BM/LiF/Al, in which triphenylamine (TPA) with thiophene (Th) moieties was investigated; however, the received value of PCE was 1.21%. In addition, the Petrus group [27] proposed an interesting small azomethine molecule, with either thiophene or benzothiazole units as its core and two TPA units at the end groups, for application in organic solar cells. For the best solar cell based on azomethine: PC_71_BM with a ratio of donors to acceptors equal to 1:5 and with PCE of 2.17% was achieved. 

On the other hand, Canli et al. [23] proposed (5-(10-undecyloxy)-2-[[[4-hexylphenyl]imino]methyl]phenol) as an acceptor in active layers, together with P3HT. The proposed azomethine exhibited liquid crystalline properties and received a value of PCE of 0.27% after annealing the active layer in the temperature of azomethine phase transition (PCE before annealing was 0.16%). Moussalem et al. [24] proposed azomethine with benzodifuran moieties and thiophene rings as a donor material, together with fullerene C_60_ used as an acceptor. The authors received PCE = 1.18%. 

As it was presented, imines were proposed as donor or acceptor components in organic solar cells. Additionally, unsymmetrical azomethine with porphyrin moieties was tested by Jeevadason et al. [26] in dye-sensitized solar cells (DSSC) and showed a value of PCE equal to 1.75%. 

The Petrus group [28] used small azomethine-based molecules with TPA units as hole-transporting materials (HTM) for perovskite solar cells, and a PCE equal to 11.9% was achieved. In all devices investigated in [28], tert-butylpyridine and lithium bis(trisfluoromethylsulfonyl)imide were added to the HTM solutions, together with tris(2-(1H-pyrazol-1-yl)pyridine)cobalt(II)bis(hexafluorophosphate) (FK102) used as a co-dopant.

In this study, four new aromatic imines were synthesized via a one-step high temperature condensation reaction. We increased the conjugated length of the electron donor moiety by introducing thiophene rings into the trimer unit of di-BenzThAz-terTh. In addition, we introduced thiazole groups in various places in synthesized imines to modify the donor–acceptor features of all compounds. The imines with thiazole moieties are proposed in this work as new small organic compounds; due to the presence of the imine group, the electron cloud of the aromatic ring and electronegative nitrogen, oxygen, and sulphur atoms should positively influence the optical properties. Moreover, triphenylamine, thiophene, or benzthiazole moieties in the investigated new imines should also provide good charge-carrier transport, causing enhancement of optical properties with the benefit of solution processability. 

UV–Vis absorption properties of synthesized imines were screened based on five main factors, as follows:

(i) influence of the positions of thiazole rings in the imines,
(ii) influence of the thiophene rings in imine,
(iii) influence of symmetry of the imines,
(iv) concentration of imines,
(v) presence of imines in compositions with PTB7.

This paper reports the first example of the use of new imines with a diverse molecular structure as components of a mixture with PTB7 to tune the absorption spectrum of active material towards future photovoltaic applications. Following the present trends in organic solar cells, new n-type materials (acceptors) or donor–acceptor materials that can replace the widely used fullerene-based derivatives are still required, taking into consideration price and some synthesis steps of PC_71_BM. 

## 2. Experimental Section

### 2.1. Materials 

All chemicals and solvents were of reagent grade and were obtained from Aldrich Chemical Co (Saint Louis, MO, USA).

### 2.2. Synthesis of Imines

#### 2.2.1. Synthesis of (N^3^ E, N^5^ E)-N ^3^, N^5^-bis(4-(di-p-Tolylamino)Benzylidene)-1,2,4-Thiadiazole-3,5-Diamine (3,5-di(diMeTPA)-1,2,4-ThdiAz)

The imine 3,5-di(diMeTPA)-1,2,4-ThdiAz [29] was obtained using a one-step high-temperature condensation reaction under reflux equipped with anhydrous CaSO_4_ as the water trap. A single-neck flask with a magnetic stir bar was filled with 4-(ditolylamino)benzaldehyde (2 mmol), 1,2,4-thiadiazole-3,5-diamine (1 mmol), *p*-toluenesulfonic acid (*p*-TS), and 12 mL of *N,N*-Dimethylacetamide (DMA). The reaction mixture was stirred for 24 h at 160 °C in an oil bath. The raw imine was precipitated in water and collected by filtration. The solid was washed with ethanol and acetone, and was recrystallized from acetone:hexane. The final imine was dried overnight at 80 °C. 

3,5-di(diMeTPA)-1,2,4-ThdiAz: Red-brown powder, yield: 45%. ^1^H NMR (400 MHz, CDCl_3_), δ [ppm]: 8.31 (2H, d, -HC=N-); 7.65–7.55 (8H, m, arom. -Ph-); 7.4–7.0 (16 H, arom. -Ph-); 2.35 (12H, t, -CH_3_). FTIR (cm^−1^): 3025 w ν(CH)_ar_, 2920 m ν_as_(CH_3_), 2855 w ν_s_(CH_3_), 2217 m, 1617 w ν(HC=N), 1597 m ν(C=C)_ar_, 1506 m δ(CCH), 1387 m δ(CCH), 1320 m ν(C-C), 1294 br ν(C=N), 1173 m δ(CCH), 814 s π(CCH)_ar_, 722 m ω(CCH)_ar_, 567 m δ(CNC), 517 s γ(CCC) (w—weak, m—medium, s—strong, br—broad, ν—stretching; δ—bending in-plane, π—bending out-of-plane).

#### 2.2.2. Synthesis of (E)-N-(4-(di-p-Tolylamino)Benzylidene)-1,3,4-Thiadiazol-2-Amine (2-diMeTPA-1,3,4-ThdiAz)

The imine 2-diMeTPA-1,3,4-ThdiAz was obtained using a one-step high-temperature condensation reaction under reflux equipped with anhydrous CaSO_4_ as the water trap. A single-neck flask with a magnetic stir bar was filled with 4-(ditolylamino)benzaldehyde (1 mmol), 2-amino-1,3,4-thiadiazole (1.25 mmol), and *p*-toluenesulfonic acid (*p*-TS). The reaction mixture was stirred for 65 h at 150 °C in an oil bath. The crude solid was dissolved in 10 mL of DMA; next, it was precipitated in water and collected by filtration. The solid was washed with ethanol and acetone, and was recrystallized twice from acetone:hexane. The final imine was dried overnight at 80 °C.

2-diMeTPA-1,3,4-ThdiAz: Red-brown powder, yield: 27%. ^1^H NMR (400 MHz, CDCl_3_), δ [ppm]: 8.54 (1H, s, -HC=N-); 7.75–7.73 (4H, m, arom. -Ph-); 7.4–7.0 (10 H, arom. -Ph); 2.23 (6H, s, aliphatic). FTIR (cm^−1^): 3025 m ν(CH)_ar_, 2919 m ν_as_(CH_3_), 2865 w ν_s_(CH_3_), 1597 s ν(C=C)_ar_, 1507 s ν(C=C)_ar_, 1321 s ν(C-C), 1295 br ν(C=N), 1180 m δ(CCH), 1109 m δ(CCH), 1033 m δ(CCH), 1017 m δ(CCH), 814 s π(CCH)_ar_, 719 m ω(CCH)_ar_, 682 m δ(NCC), 567 m δ(CNC), 518 m γ(CCC) (w—weak, m—medium, s—strong, br—broad,ν—stretching; δ—bending in-plane, π—bending out-of-plane, γ—torsion).

#### 2.2.3. Synthesis of (N,N′E,N,N′E)-N,N′-([2,2′:5′,2″-Terthiophene]-5,5″-Diylbis(Methanylylidene))bis(Benzo[d]Thiazol-2-Amine (di-BenzThAz-terTh)

The imine di-BenzThAz-terTh [30] was obtained using a one-step high-temperature condensation reaction under reflux equipped with anhydrous CaSO_4_ as the water trap. A single-neck flask with a magnetic stir bar was filled with 2,2′:5′,2″-terthiophene-5,5″-dicarboxaldehyde (2 mmol), 2-aminobenzothiazole (5 mmol), and p-toluenesulfonic acid, which were then mixed. The crude solid was dissolved in 10 mL of DMA and was next precipitated in water and collected by filtration. The solid was washed with ethanol and acetone, and was next washed with hot acetone under stirring. The final imine was dried overnight at 80 °C.

di-BenzThAz-terTh: Dark-brown powder, yield: 17%. ^1^H NMR (400 MHz, CDCl_3_), δ [ppm]: 9.31 (2H, s, -HC=N-); 7.87–8.04 (4H, dd, benzothiazole, positions 4 and 7); 7.4 (4 H, m, benzothiazole, positions 5 and 6); 7.27 (2H, s, terthiphene, positions 4 and 4″); 7.16 (2H, t, terthiphene, positions 3′ and 4′); 6.95 (2H, t, terthiphene, positions 3 and 3″). FTIR (cm^−1^): 3060 ν(CH)_ar_, 1659 s, 1587 ν(HC=N), 1570 ν(C=C)_ar_, 1545 ν(C=C)_ar_, 1489 δ(C_ar_CH), 1443, 1422, 1334, 1313, 1253, 1229, 1218, 1151 δ(CH)_ar_ ip, 1056 δ(CH)_ar_ ip, 1014, 972, 909, 884, 867 ω(CCH)_ar_, 796, 777, 755 ω(CCH)_ar_, 749, 725, 720, 667, 593, 555, 5030, 476, 434. (w—weak, m—medium, s—strong, br—broad, ν—stretching; δ—bending in-plane, π—bending out-of-plane, γ—torsion).

#### 2.2.4. Synthesis of 4-((E)-((4-(4-(4-Fluorophenyl)Thiazol-2-yl)Phenyl)Imino)Methyl)-N-(4-((E)-((4-(4-(4-Fluorophenyl)Thiazol-2-yl)Phenyl)Imino)Methyl)Phenyl)-N-Phenylaniline (di(FPh-ThAz-An)-TPA)

The imine di(FPh-ThAz-An)-TPA [31] was obtained using a one-step high-temperature condensation reaction under reflux equipped with anhydrous CaSO_4_ as the water trap. A single-neck flask with a magnetic stir bar was filled with 4,4′-diformyltriphenylamine (2 mmol), 4-[4-(4-fluorophenyl)1,3-thiazol-2-yl]aniline (5 mmol), and p-toluenesulfonic acid, which were then mixed. Finally, the solid was dried for 24 h at 60 °C. 

di(FPh-ThAz-An)-TPA: Brown powder, yield: 32%.^1^H NMR (400 MHz, CDCl_3_), δ [ppm]: 8.48 (2H, s, -HC=N-); 7.81–7.70 (12H, m, arom. -Ph-); 7.45–7.05 (17 H, arom. -Ph, -Ph-); 6.70 (2H, d, tiazol). FTIR (cm^−1^): 3031 w ν(CH)_ar_, 1623 w ν(HC=N), 1585 vs. ν(C=C)_ar_, 1504 vs. ν(C=C)_ar_, 1480 vs. δ(C_ar_CH), 1430 w, 1410 w, 1318 m, 1288 m, 1220 m, 1197 w, 1165 s δ(CH)_ar_, 1111 w δ(CH)_ar_, 1098 w, 1056 m, 1032 w δ(CH)_ar_, 1012 m, 981 s, 901 m, 884 m, 840 s ω(CCH)_ar_, 751s ω(CCH)_ar_, 696 s, 595 m, 571 s, 540 s, 516 m. (w—weak, m—medium, s—strong, vs—very strong, ν—stretching; δ—bending in-plane, π—bending out-of-plane, γ—torsion).

### 2.3. Characterization of Methods

The imines were characterized using several techniques. Samples were characterized with ^1^H NMR, using deuterated chloroform (CDCl_3_) as a solvent and with a Jeol ECZ-400 S spectrometer (Akishima, Tokyo, Japan) (^1^H, 400 MHz) with a delay time of 5 s. Measurements were carried out at room temperature on 10–15% (w/v) sample solutions. 

The Fourier-transform mid-infrared (MIR) spectra of the imines in the region of 4000–400 cm^−1^ were measured at 2 cm^−1^ resolution with co-addition of 32 scans on a Nicolet-Nexus spectrometer (Farmingdale, NJ, United States) using the KBr pellet technique. 

Absorption spectra of imines and imine: PTB7 compositions in 1,2-dichlorobenzene (DCB) were recorded in the range from 290 nm to 800 nm using the spectrometer Agilent Cary 300 (Agilent Technologies, Santa Clara, United States).

Calibration curves for imine and the mixture of PTB7 and imine were made. For each sample, 5 dilutions were made containing different proportions of the imine starting solution—20%, 40%, 60%, 80%, and 100%—and the leftover volume was filled with DCB. In the case of the PTB7:imine mixtures, the calibration was done with 5 solutions, always maintaining the same concentration of polymer and the same total concentration of imine as for experiments with imine alone. DCB was used as a blank sample.

PTB7 (0.0012 g) was solubilized in 50 cm^3^ of DCB (c = 2.48 × 10^−5^ M) after stirring with heating at 50–65 °C during 5 h. 

Thermogravimetric analysis (TG-DTA) and Differential Scanning Calorimetry (DSC) were performed on a LABSYS EVO 1150 °C TG-DTA/DSC instrument (Caluire, France). Samples (approximately 3–5 mg) were contained in alumina pans in the presence of nitrogen as the furnace atmosphere. Measurements were performed from ambient temperature up to 800 °C with a heating rate of 5 °C/min.

Theoretical calculations, including the highest occupied molecular orbital (HOMO) and the lowest unoccupied molecular orbital (LUMO) energies of the compounds and vibration spectra (FTIR), were performed using the Gaussian 16 software package (Gaussian, Inc., Wallingford CT, United States) [32]. The geometries of the isolated imines were fully optimized using density functional theory (DFT) with the B3LYP hybrid functional and the 6-31G(d,p) basis set [33,34,35,36]. The absence of negative frequencies confirmed that the stationary point on the potential surface in the geometry optimization was achieved.

Thermal behavior was conducted as described elsewhere [37]; infrared images were recorded using thermographic camera (VIGOcam v50, VIGO System S.A, Poland) for sandwich-like samples as a response to a bias voltage between 0 and 10 V, applied using a multichannel potentiostat-galvanostat (PGStat Autolab M101, Metrohm, Nederland) connected to computer. The active area of the constructed devices was approximately 1.5 cm^2^ in all cases.

## 3. Results and Discussion

In this work, we aim at the synthesis of new thermally stable organic compounds—imines—and their potential application in organic solar cells. The main goal of this work was proving that it is possible to design and obtain an optimal ratio of imine to PTB7 for polymer solar cells through the deep investigation of various donor–acceptor mixtures through UV–Vis studies. 

The molecular concept of the four new imines that were investigated is based on the following known structural aspects:

✓ The presence of triphenylamine, thiophene, or benzothiazole moieties in the imine structure should enhance hole transport properties and optical properties in the UV–Vis range.
✓ The presence of F atoms in imine should cause a combination of polar and steric effects and confirms stability on fluoro-substituted imine due to the great strength of the C–F bond.
✓ The combination of these structural scaffolds gives us the possibility to create new imines with the benefit of solution processability.

In this work, we determined the impact of the chemical constitution of an imine and the volume composition with PTB7 of an organic blend on the optical, electrical, and thermal characteristics.

In connection with the above, four new aromatic imines with thiazole moieties were synthesized and investigated in depth by UV–Vis spectroscopy in the solution. The synthetic routes of the investigated compounds are presented in Figure 1. The newly designed amines were to answer the question of whether imines with properly selected structures can be useful for tuning the absorption spectra of poly({4,8-bis[(2-ethylhexyl)oxy]benzo[1,2-b:4,5-b′]dithiophene-2,6-diyl}{3-fluoro-2-[(2-ethylhexyl)carbonyl]thieno[3,4-b]thiophenediyl} (PTB7) by filling the weak absorption area, as well as choosing the concentrations of individual imines relative to PTB7. 

**Figure 2:**
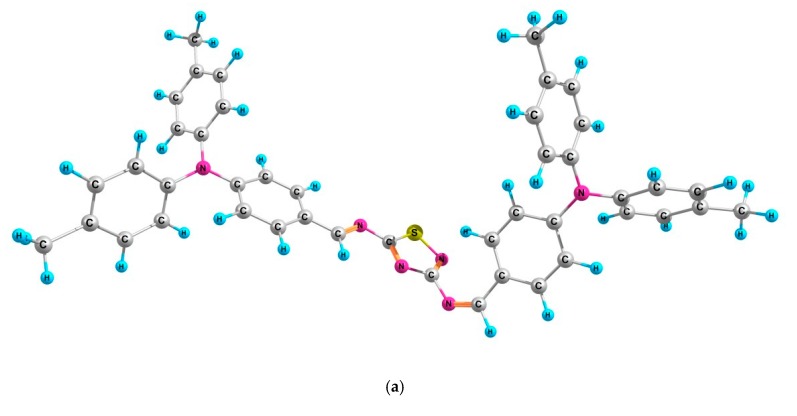

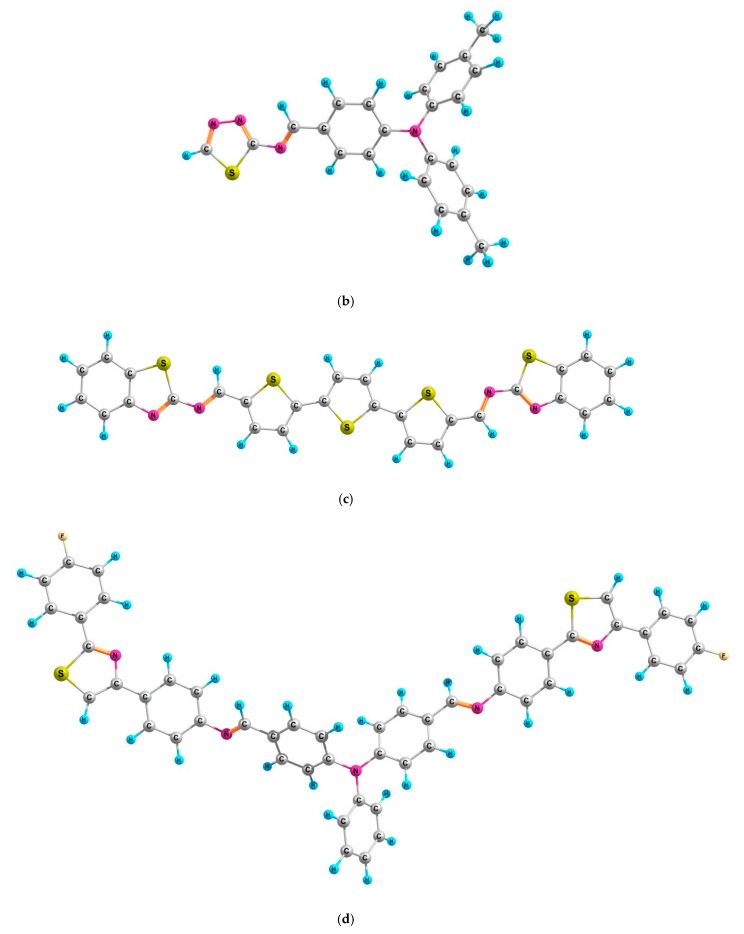
The optimized structures of the investigated imines (**a**) 3,5-di(diMeTPA)-1,2,4-ThdiAz, (**b**) 2-diMeTPA-1,3,4-ThdiAz (**c**) di-BenzThAz-terTh (**d**) di(FPh-ThAz-An)-TPA.

The optimized chemical structures for all imines are shown in Figure 2, and details or theoretical calculations are presented in the Supporting Information. The LUMO–HOMO gap of imines was calculated to be in the range 2.85–3.57 eV (see Table S1).

**Figure 3:**
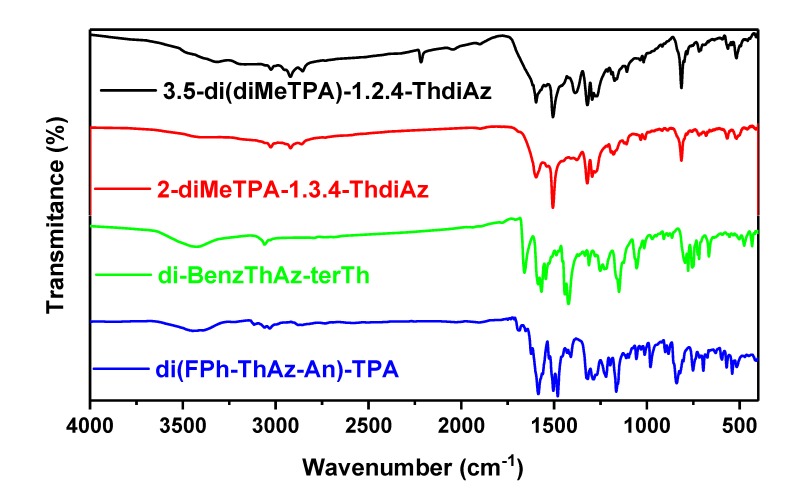
FTIR spectra of the investigated imines.

Details of synthesis procedures and molecular characteristics along with proton NMR and FTIR spectra of the imines are described in detail in the Experimental Section. The absence of the residual amino (NH_2_) and aldehyde (CHO) groups, together with the appearance of a band typical of an imine bond (HC=N-), was confirmed by the FTIR and ^1^H NMR spectra. In the proton NMR spectra of the investigated compounds, the imine proton signal was observed in the range of 8.31–9.31 ppm, and its position depended on the chemical structure of the investigated imine (see Experimental Section). The presence of the imine group was also confirmed by FTIR spectroscopy, since in each case, the band characteristic of the HC=N- stretching vibrations was detected. The exact position of this band varies in the spectral range of about 1600–1659 cm^−1^ (see Figure 3).

The calculations of vibrational frequencies of all investigated imines were performed with the B3LYP hybrid functional and the 6-31G(d,p) basis set, as is presented in the Supporting Information (Figures S1–S4). The theoretical data obtained from FTIR spectroscopy were quite compatible with the experimental data. For example, as seen in Figure 2, the band at 1617 cm^−1^ corresponds to *ν*(C=N) in 3,5-di(diMeTPA)-1,2,4-ThdiAz; imine was calculated as 1621 cm^−1^. For di(FPh-ThAz-An)-TPA, an imine band was found at 1623 cm^−1^, while theoretical study showed this band at 1636 cm^−1^. 

**Figure 4:**
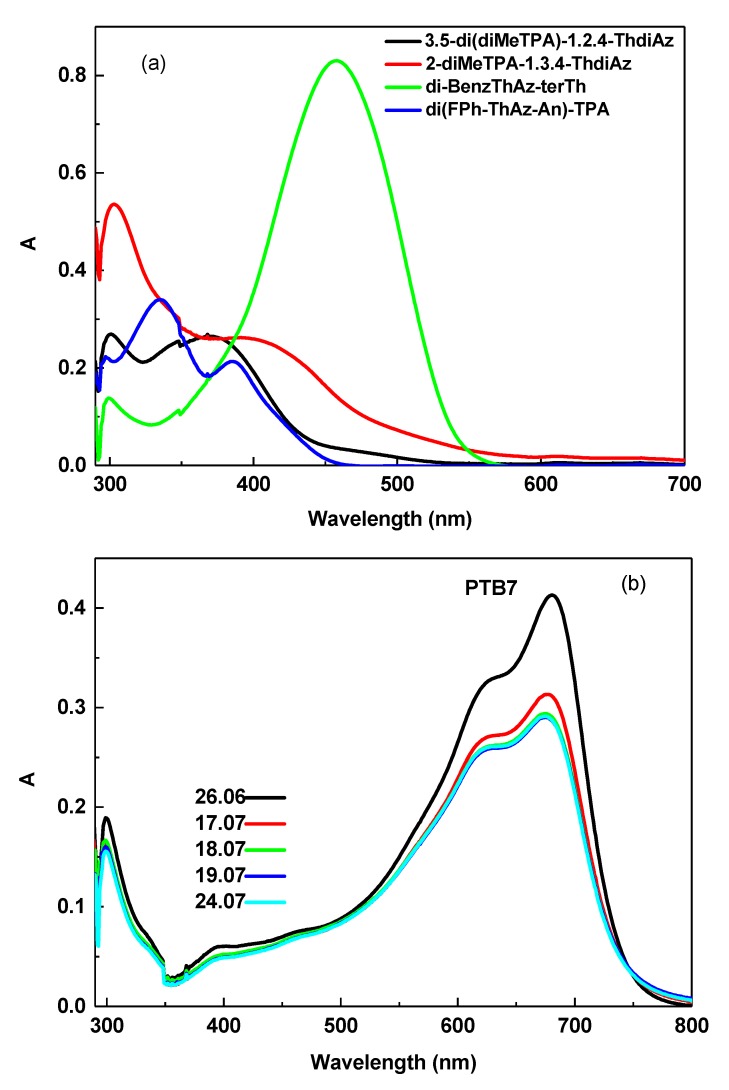
UV–Vis spectra of (**a**) imines and (**b**) PTB7 in 1,2-dichlorobenzene (DCB).

The optical properties of imines in 1,2-dichlorobenzene (DCB) were investigated by UV–Vis absorption spectroscopy. The UV–Vis absorption spectra of the imines are shown in Figure 4a. 

The solution of the investigated di-BenzThAz-terTh imine showed one absorption band with a maximum peak at 457 nm, while other imines presented two absorption bands with maximum peaks at 290–333 nm and 375–400 nm (see Figure 4a). In the DCB solution, di-BenzThAz-terTh exhibited a red shift of the absorption band maximum (responsible for the π–π* transition in the imine group) in comparison with the other investigated imines caused by the presence of thiophene rings in this compound. On the other hand, taking into consideration the symmetry of the investigated imines, i.e., 3,5-di(diMeTPA)-1,2,4-ThdiAz and 2-diMeTPA-1,3,4-ThdiAz, we can conclude that the symmetrical imine 3,5-di(diMeTPA)-1,2,4-ThdiAz exhibited a blue shift of about 25 nm compared with 2-diMeTPA-1,3,4-ThdiAz. For the investigated PTB7 polymer, we observed three main absorption bands at 300, 621, and 680 nm, as is shown in Figure 4b. Taking into consideration the fact that, for polymers applied in solar cells, stability in time is the main factor, we thus investigated UV–Vis optical properties of PTB7 in DCB over a period of about one month. As it is presented in Figure 4b, the PTB7 after about 20 days exhibited a hypochromic effect in comparison with the pristine sample. For this reason, we investigated only freshly prepared mixtures of PTB7 with imines. 

The thermal properties of the four investigated imines were characterized by both differential scanning calorimetry (DSC) and thermogravimetric analysis (TGA). Our study showed that the melting points of the imines increased as follows: 145 °C (di(FPh-ThAz-An)-TPA) < 153 °C (2-diMeTPA-1,3,4-ThdiAz) < 252 °C (3,5-di(diMeTPA)-1,2,4-ThdiAz) < 287 °C (di-BenzThAz-terTh). The highest melting point was found for the symmetrical imine with thiophene moieties, while the lowest one for the symmetrical imine with F atoms.

The TGA curve of imine indicates one main reaction stage. The initial decomposition based on 5% weight loss occurred in the range of 280–330 °C, depending on the structure of the imine. 

The decomposition at 800 °C was defined as the char yield percentage at about 86% for the di-BenzThAz-terTh and di(FPh-ThAz-An)-TPAimines. On the other hand, the 3,5-di(diMeTPA)-1,2,4-ThdiAz and 2-diMeTPA-1,3,4-ThdiAz imines showed lower values of the char yield percentage compared with the other two imines (30% and 68%, respectively). The TGA analysis suggested that the investigated imines had good thermal stability in the nitrogen atmosphere.

### 3.1. UV–Vis Study of 3,5-di(diMeTPA)-1,2,4-ThdiAz and PTB7:3,5-di(diMeTPA)-1,2,4-ThdiAz Composition

**Figure 5:**
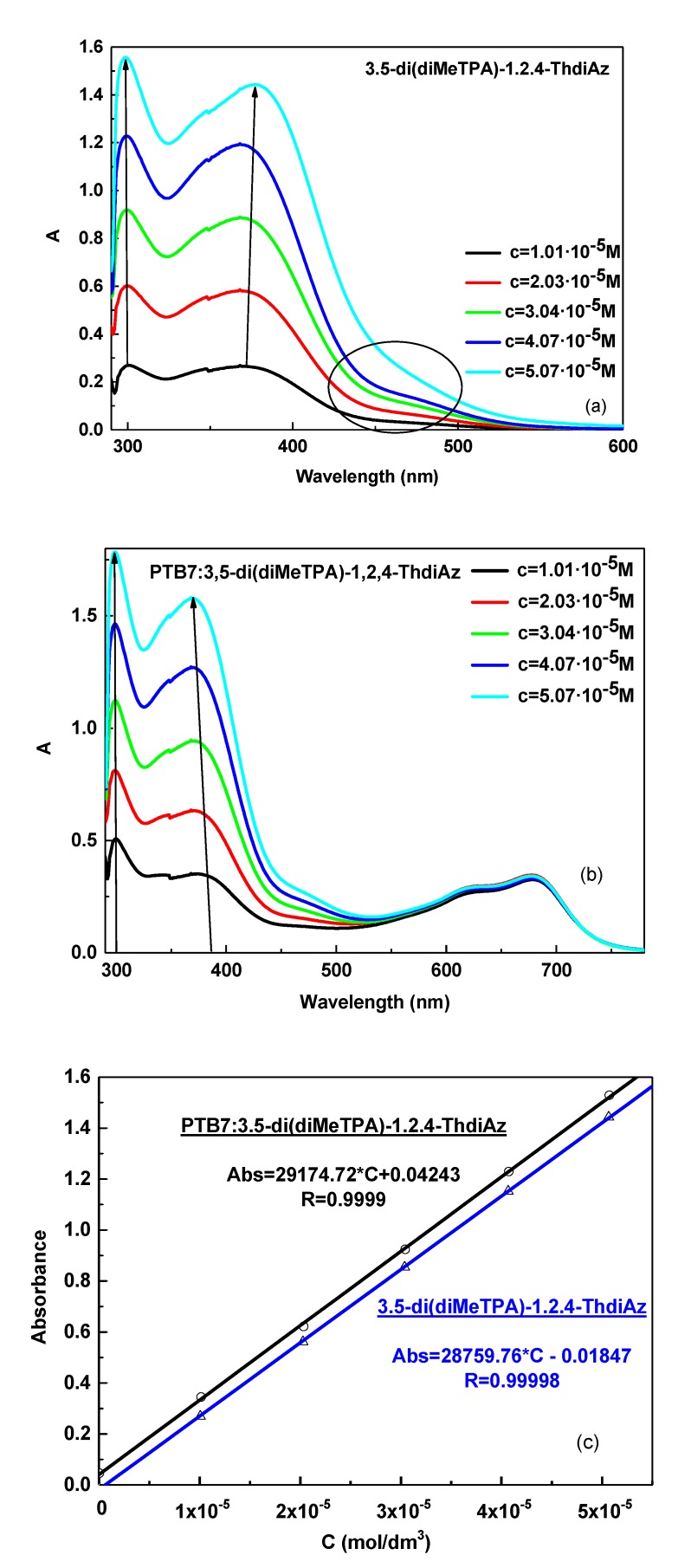
Concentration-dependent solution UV–Vis spectra of the (**a**) 3,5-di(diMeTPA)-1,2,4-ThdiAz, (**b**) PTB7: 3,5-di(diMeTPA)-1,2,4-ThdiAz in DCB solution (conc. increase from 1 × 10^−5^ to 5 × 10^−5^ M), together with (**c**) calibration curves for 3,5-di(diMeTPA)-1,2,4-ThdiAz and PTB7: 3,5-di(diMeTPA)-1,2,4-ThdiAz.

In this part of our work, we investigated the symmetrical imine with the thiazole ring in the core and two triphenylamine (TPA) moieties as the terminal group or groups. As is shown in Table 1 and in Figure 5a, the 3,5-di(diMeTPA)-1,2,4-ThdiAz imine was studied in DCB by UV–Vis spectroscopy at various concentrations, monitoring the changes in the π–π* and n–π* transitions. Our study showed that along with increased concentration of 3,5-di(diMeTPA)-1,2,4-ThdiAz from 1 × 10^−5^ to 5 × 10^−5^ M, we did not observe changes in the maximum of the absorption band (λ_max_). Some changes in UV–Vis absorption spectra of 3,5-di(diMeTPA)-1,2,4-ThdiAz were observed in the range of 420–500 nm as a broad shoulder along with increased concentration (see Figure 5a—part in circle). This behavior can probably be explained by the fact that along with an increasing concentration of 3,5-di(diMeTPA)-1,2,4-ThdiAz in DCB, the molecules changed their conformations. The molar absorption coefficient (*ε*) of 3,5-di(diMeTPA)-1,2,4-ThdiAz was found in the range of 28010–28968 M^−1^ cm^−1^ for the imine bond, as is presented in Table 1. Moreover, in the UV–Vis spectra of the 3,5-di(diMeTPA)-1,2,4-ThdiAz, the hyperchromic effect along with increasing concentration was observed.

The addition of 3,5-di(diMeTPA)-1,2,4-ThdiAz in PTB7 resulted in a small blue shift of the imine bond together with the hyperchromic effect observed along with increase of concentration (see Figure 5b). It was curious that the absorption bands in the range of 550–750 nm in the PTB7: 3,5-di(diMeTPA)-1,2,4-ThdiAz composition did not change in intensity along with the increasing imine concentration. The molar absorption coefficients of PTB7: 3,5-di(diMeTPA)-1,2,4-ThdiAz are presented in Table 1.

**Figure 6:**
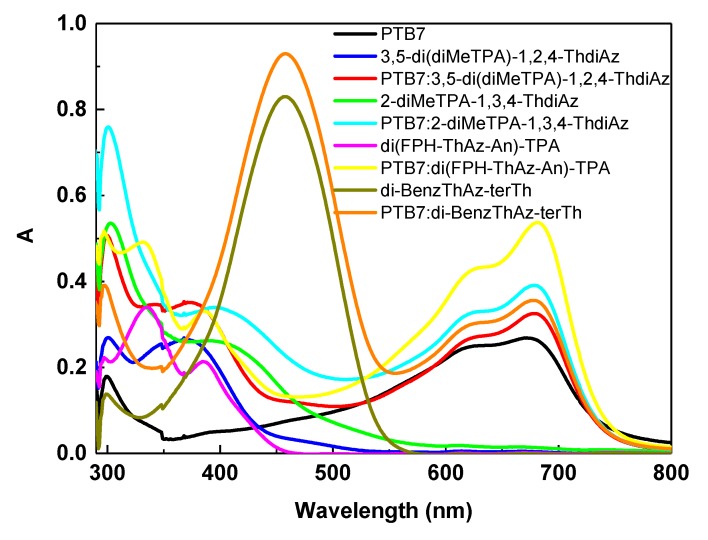
UV–Vis spectra of all imines and PTB7:imine in DCB with isosbestic points.

For the 3,5-di(diMeTPA)-1,2,4-ThdiAz and PTB7, one isosbestic point was found at about 440 nm (Figure 6), appearing as an effect of the interactions between both compounds. 

### 3.2. UV–Vis Study of 2-diMeTPA-1,2,4-ThdiAz and PTB7: 2-diMeTPA-1,2,4-ThdiAz Composition

**Figure 7:**
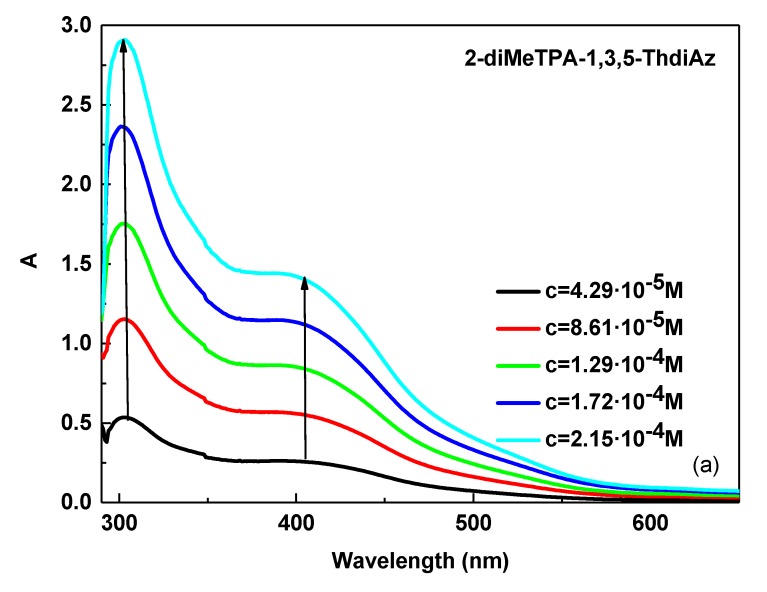

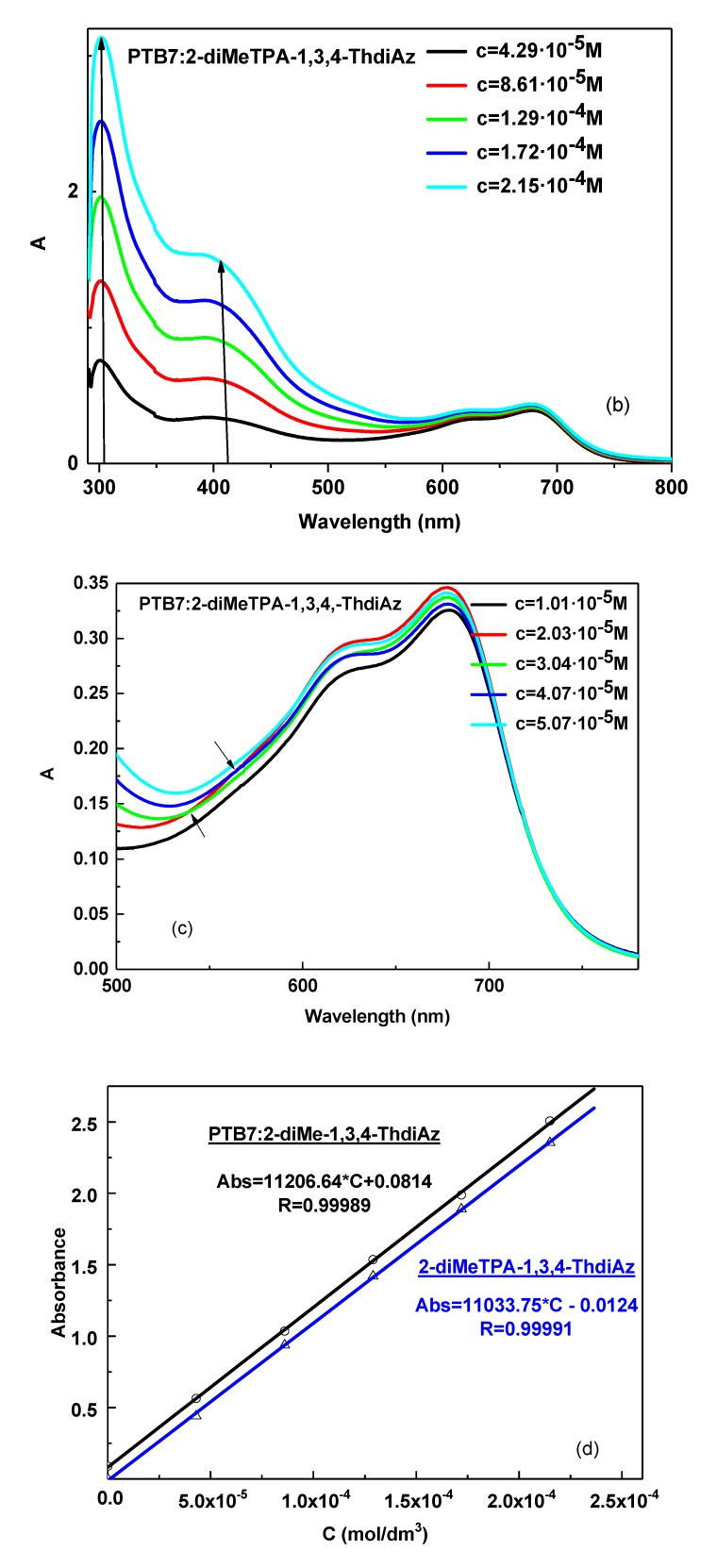
Concentration-dependent solution UV–Vis spectra of the (**a**) 2-diMeTPA-1,3,4-ThdiAz, (**b**,**c**) PTB7: 2-diMeTPA-1,3,4-ThdiAz in DCB solution (conc. increase from 4.29 × 10^−^^5^ to 2.15 × 10^−^^4^ M), together with (**d**) calibration curves for 2-diMeTPA-1,3,4-ThdiAz and PTB7: 2-diMeTPA-1,3,4-ThdiAz.

In the next step of our work, we analyzed how imine symmetry affects their absorption properties in the UV–Vis range. For this reason, we synthesized the unsymmetrical imine 2-diMeTPA-1,2,4-ThdiAz with thiazole and the TPA unit and compared its selected absorption properties with those of the symmetrical 3,5-di(diMeTPA)-1,3,4-ThdiAz imine. As presented in Figure 7a and Table 2, imine 2-diMeTPA-1,2,4-ThdiAz exhibited a behavior in the DCB solution with increasing concentration similar to that of 3,5-di(diMeTPA)-1,3,4-ThdiAz, taking into consideration the intensity and position of the investigated absorption bands. The molar absorption coefficient (ε) of 2-diMeTPA-1,2,4-ThdiAz was found at the lower value compared with that of 3,5-di(diMeTPA)-1,3,4-ThdiAz imine, and was in the range of 6140–6777 M^−1^ cm^−1^ for the imine bond, as presented in Table 2. 

The addition of 2-diMeTPA-1,3,4-ThdiAz in PTB7 similar results to those of the case of 3,5-di(diMeTPA)-1,2,4-ThdiAz in a small blue shift of the imine band together with an observed hyperchromic effect with the increasing imine concentration of 2-diMeTPA-1,3,4-ThdiAz in the DCB solution (see Figure 7b). Opposite behavior to PTB7: 3,5-di(diMeTPA)-1,2,4-ThdiAz was found for the PTB7: 2-diMeTPA-1,3,4-ThdiAz composition in the range of 500–750 nm (see Figure 7c). In this absorption range, the intensities of two absorption bands changed along with the increase of the imine concentration. Moreover, it can be seen that two isosbestic points (indicated in arrows in Figure 7c) were found. The molar absorption coefficients (ε) of PTB7: 2-diMeTPA-1,3,4-ThdiAz are presented in Table 2. This behavior suggested that the symmetry of imines influences the UV–Vis absorption properties of PTB7. 

For the 2-diMeTPA-1,3,4-ThdiAz and PTB7, one isosbestic point at about 495 nm was found (Figure 6), and had a red shift of about 55 nm compared with the PTB7 and 3,5-di(diMeTPA)-1,2,4-ThdiAz.

### 3.3. UV–Vis Study of di(FPh-ThAz-An)-TPA and PTB7: di(FPh-ThAz-An)-TPA Compositions

**Figure 8:**
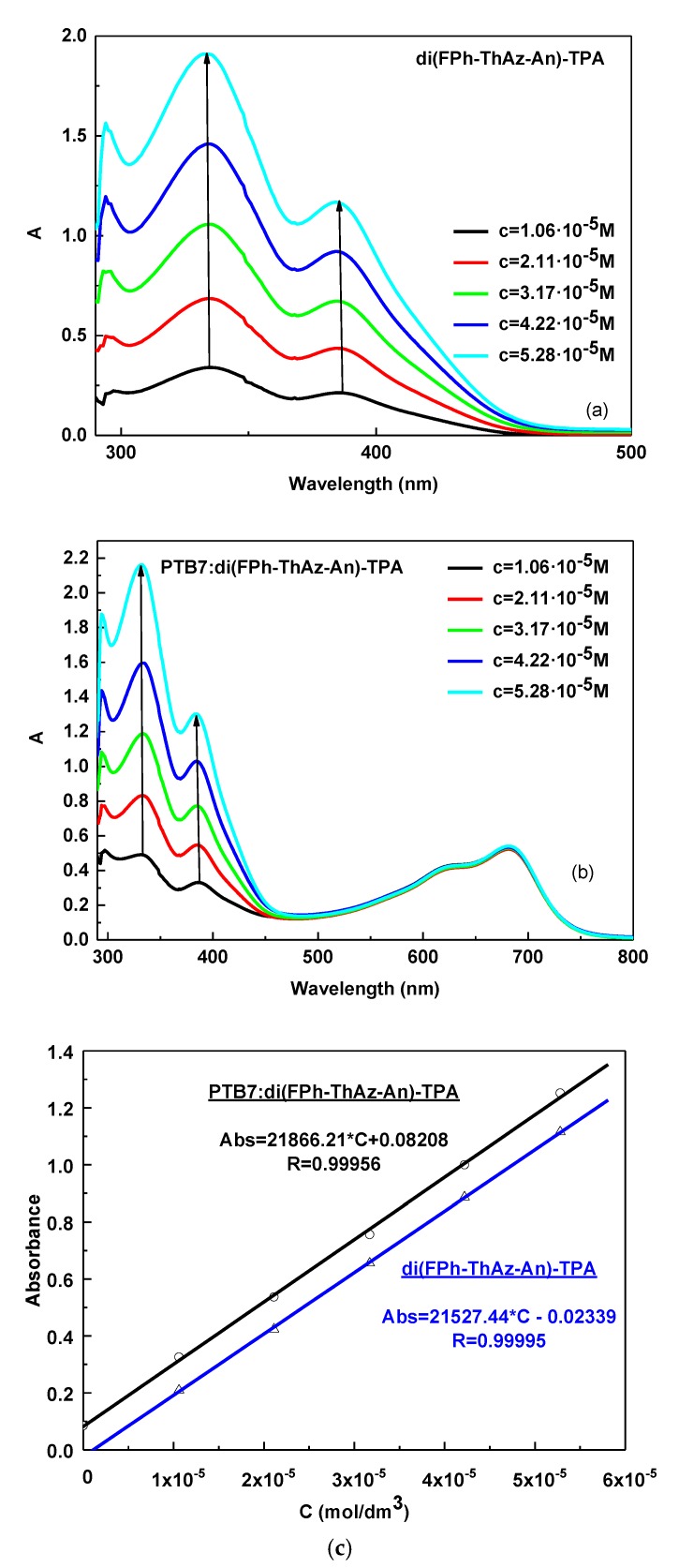
Concentration-dependent solution UV–Vis spectra of the (**a**) di(FPh-ThAz-An)-TPA, (**b**) PTB7: di(FPh-ThAz-An)-TPA in DCB solution (conc. increase from 1.06 × 10^−^^5^ to 5.28 × 10^−^^5^ M), together with (**c**) calibration curves for di(FPh-ThAz-An)-TPA and PTB7: di(FPh-ThAz-An)-TPA.

It is well known that the placement of functional groups in compounds influences also their properties. In this part of our work, we investigated a symmetrical imine with a TPA unit in the core and thiazole and fluorene moieties at the end of di(FPh-ThAz-An)-TPA by UV–Vis spectroscopy. The obtained results are summarized in Table 3 and Figure 8. The Imine di(FPh-ThAz-An)-TPA exhibited two main absorption bands at 333 nm and 385 nm in the DCB solution. Along with increasing concentration of di(FPh-ThAz-An)-TPA, the intensity of the investigated absorption bands increased, while the maxima of absorption bands were not shifted. The molar absorption coefficient (ε) of di(FPh-ThAz-An)-TPA was found in the range of 19962–22294 M^−^^1^ cm^−^^1^ for the imine bond, as presented in Table 3. 

The addition of di(FPh-ThAz-An)-TPA to PTB7 did not change the position of any absorption bands in the range of 290–800 nm. Similarly, as in the case of the symmetrical imine 3,5-di(diMeTPA)-1,2,4-ThdiAz, a hyperchromic effect in the PTB7:di(FPh-ThAz-An)-TPA composition was observed for the absorption bands corresponding to imine structure in the range 300–450 nm, detected along with increasing imine concentration (see Figure 8b). On the other hand, the absorption bands in the range of 550–800 nm assigned to the PTB7: di(FPh-ThAz-An)-TPA composition did not change polymer intensity with increasing imine concentration, as in the case of PTB7: 3,5-di(diMeTPA)-1,2,4-ThdiAz. The molar absorption coefficients of PTB7: di(FPh-ThAz-An)-TPA are presented in Table 3.

The UV–Vis absorption spectra of di(FPh-ThAz-An)-TPA and PTB7 showed one isosbestic point at about 440 nm (Figure 6), similarly to the case of PTB7 and 3,5-di(diMeTPA)-1,2,4-ThdiAz.

### 3.4. UV–Vis Study of di-BenzThAz-terTh and PTB7: di-BenzThAz-terTh Compositions

**Figure 9:**
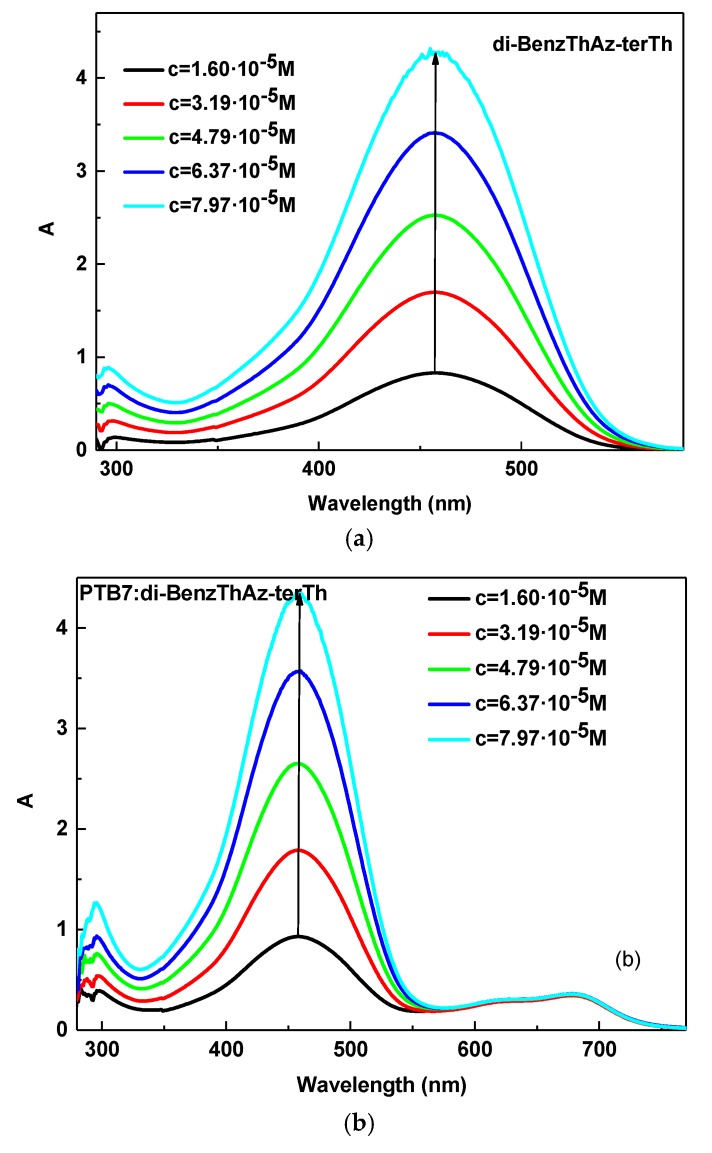

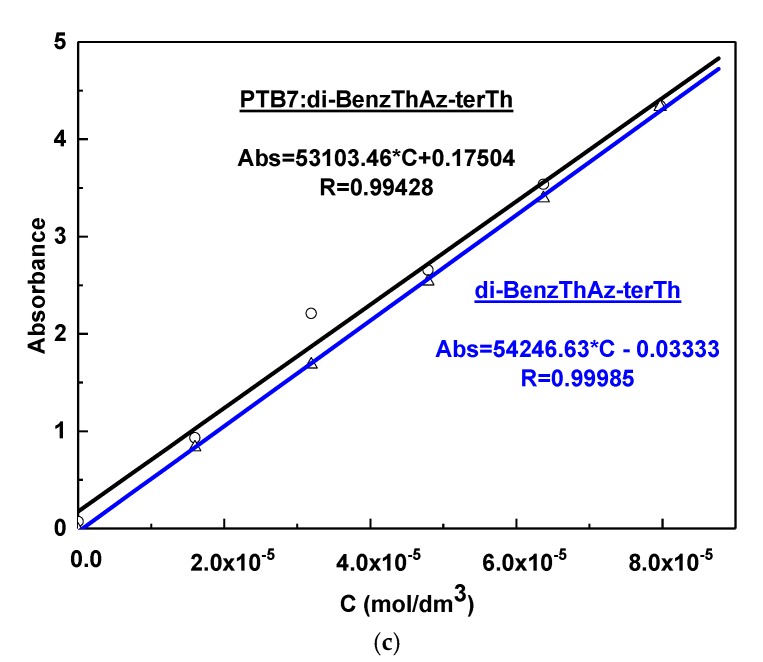
Concentration-dependent solution UV–Vis spectra of the (**a**) di-BenzThAz-terTh, (**b**) PTB7: di-BenzThAz-terTh in DCB solution (conc. increase from 1.60 × 10^−^^5^ to 7.97 × 10^−^^5^ M), together with (**c**) calibration curves for di-BenzThAz-terTh and PTB7: di-BenzThAz-terTh.

Finally, we investigated the symmetrical imine with thiazole and thiophene moieties (di-BenzThAz-terTh) by UV–Vis spectroscopy. For the di-BenzThAz-terTh imine, we can conclude that along with increasing concentration, no changes in the positions of the absorption band were found. However, similarly to all other imines investigated in this work, with increased concentration of di-BenzThAz-terTh, the intensity of the investigated absorption band increased (see Figure 9a). The molar absorption coefficient of di-BenzThAz-terTh was found in the range of 50,638–53,395 M^−1^ cm^−^^1^ for the imine bond, and was the highest for all investigated imines in this work (Table 4).

Addition of di-BenzThAz-terTh in PTB7 did not change the positions of any absorption bands; however, a hyperchromic effect in the PTB7: di-BenzThAz-terTh composition was detected (see Figure 9b). The absorption bands in the range of 550–800 nm in the PTB7: di-BenzThAz-terTh composition did not change intensity with increasing imine concentration. The molar absorption coefficients of PTB7: di-BenzThAz-terTh are presented in Table 4.

For the di-BenzThAz-terTh and PTB7, one isosbestic point was found at about 540 nm (Figure 6), and had a red shift of about 100 nm compared with the PTB7 and 3,5-di(diMeTPA)-1,2,4-ThdiAz.

### 3.5. Structural Defects in Organic Devices Based on Imine and PTB7:Imine Compositions

The thermal behavior of organic layers composed of imine or PTB7:imine was recorded for devices composed of indium tin oxide (ITO), using conductive glass as electrodes. In order to induce heating to the sample, an external potential was applied. The weight ratio between PTB7 and the corresponding imine was set according to the UV–Vis calibration experiment and is indicated in brackets. The devices’ architecture was as follows:

▪ ITO/3,5-di(diMeTPA)-1,2,4-ThdiAz/Ag/ITO,
▪ ITO/2-diMeTPA-1,3,4-ThdiAz/Ag/ITO,
▪ ITO/di-BenzThAz-terTh/Ag/ITO,
▪ ITO/di(FPh-ThAz-An)-TPA/Ag/ITO,
▪ ITO/PTB7:3,5-di(diMeTPA)-1,2,4-ThdiAz(1:0.3)/Ag/ITO,
▪ ITO/PTB7:2-diMeTPA-1,3,4-ThdiAz(1:0.7)/Ag/ITO,
▪ ITO/PTB7: di-BenzThAz-terTh(1:0.4)/Ag/ITO,
▪ ITO/PTB7:di(FPh-ThAz-An)-TPA(1:0.4)/Ag/ITO.

**Figure 10:**
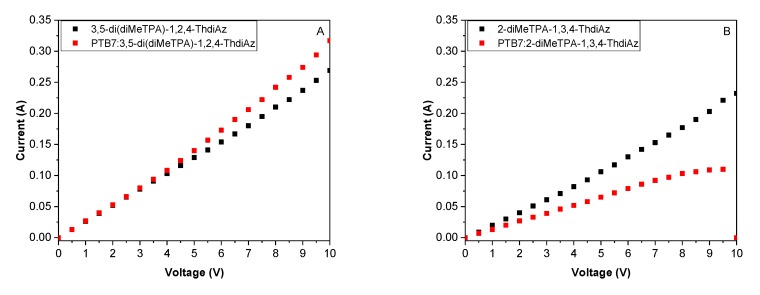

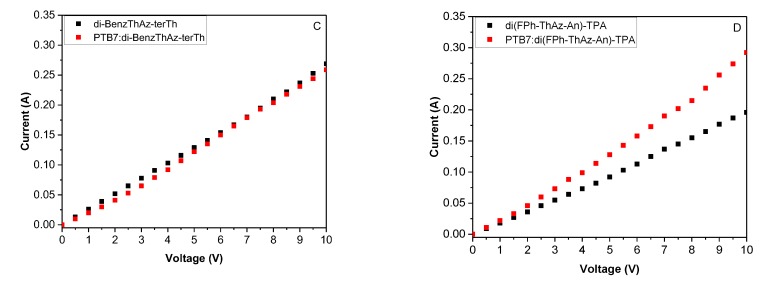
The correlation of current versus applied potential for the constructed devices containing imines and PTB7:imine mixtures.

Figure 10 presents the current flow induced by external potential applied on the sample. Generally, all samples, both the imines alone and their mixtures with PTB7, displayed linear-like tendencies upon increasing potential value. The resistance values calculated for 1 cm^2^ gave values which ranged from 21.3 to 51.3 Ω for PTB7: 3,5-di(diMeTPA)-1,2,4-ThdiAz (1:0.4) and PTB7:2-diMeTPA-1,3,4-ThdiAz (1:0.7). The presence of PTB7 in the organic layer in the case of di-BenzThAz-terTh had a neglectable influence on the current conductivity compared to that of di-BenzThAz-terTh, giving values of 25.1 and 25.2 Ω, respectively (Figure 10C). For two imines, namely for di(FPh-ThAz-An)-TPA and 3,5-di(diMeTPA)-1,2,4-ThdiAz, the presence of PTB7 in the casted mixture caused a reduction of resistance from 33.7 to 23.0 Ω and from 25.2 to 21.3 Ω, respectively (Figure 10A,D). In the last case (2-diMeTPA-1,3,4-ThdiAz), the performance of the mixture was inferior compared to that of imine alone: The resistance almost doubled, from 28.8 to 51.3 Ω (Figure 10B). Moreover, for PTB7:2-diMeTPA-ThdiAz (1:0.7), at higher voltages of above 8.0 V, a decrease of current flow to 0.0 A at 10.0 V was noted. The current decline could be related to degradation of the organic layer.

**Figure 11:**
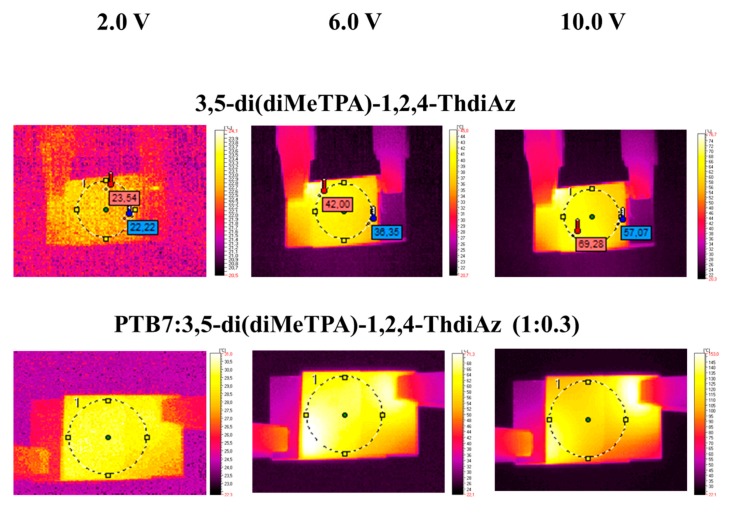

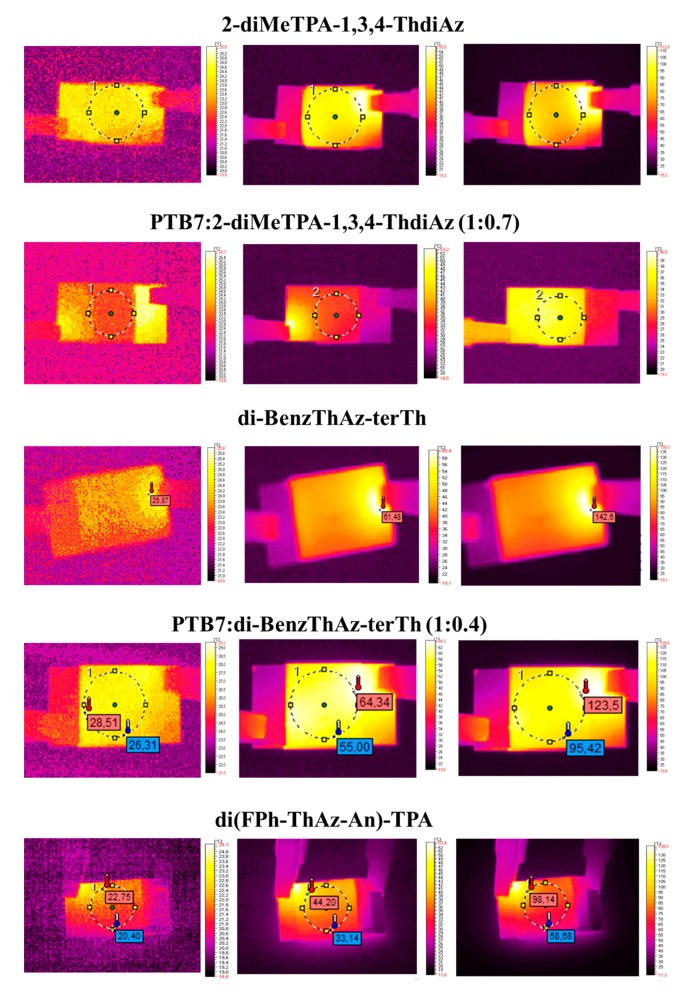

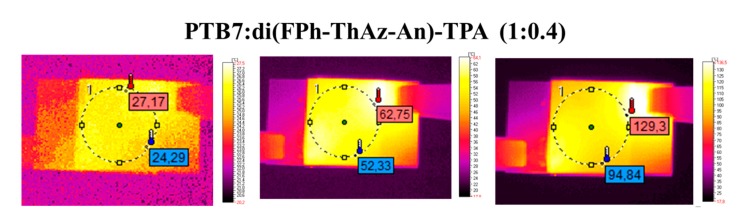
IR images obtained for device architecture: Indium tin oxide (ITO)/organic layer/Ag/ITO at 2.0 V, 6.0 V, and 9.0 V; the composition of the organic layer is listed on the left side.

The thermal images of the studied samples are displayed in Figure 11. As it can be observed, a general tendency regarding the heat distribution can be observed. Namely, the presence of PTB7 improves homogenous distribution of heat over all active areas, with the exception of PTB7:2-diMeTPA-ThdiAz (1:0.7). It was evident in the case of di(FPh-ThAz-An)-TPA and di-BenzThAz-terTh, where the organic layer contained only the imine, the heat distribution was mainly concentrated close to the metal clamps. The overheating of areas close to electrodes was also observed in our previous works [36,37]. This phenomenon might be related to the concentration of the current flow close to the metallic clamps, due to the imperfect interface between the metal and the ITO, and/or the low thermal conductivity of the organic layer.

**Figure 12:**
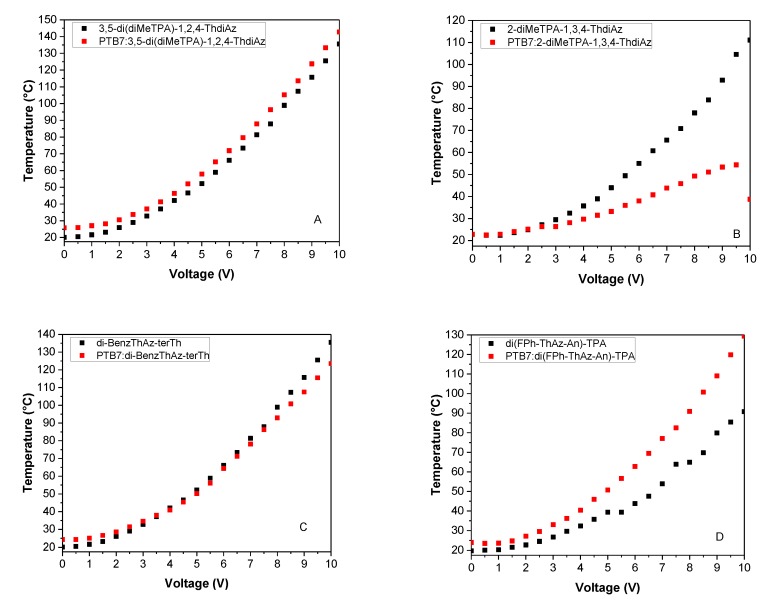
The correlation of temperature versus applied potential for the constructed devices containing imines and the PTB7:imine mixture.

The maximal thermal response observed for the studied devices ranged from 54 to 143 °C (see Figure 12). The thermal response corresponded to the current flow of these samples: The lowest temperature was observed for the mixture PTB7:2-diMeTPA-ThdiAz (1:0.7), for which a possible degradation of the organic layer was also observed as a decline of current flow. On the other hand, the highest observed temperature was for PTB7:3,5-di(diMeTPA)-1,2,4-ThdiAz. The thermal behavior aligned very similarly to the current flow across the samples: 3,5-di(diMeTPA)-1,2,4-ThdiAz, di-BenzThAz-terTh and their mixtures with PTB7 gave almost identical thermal images with very similar maximal temperatures, valued at approximately 140 and 135 °C, respectively. A higher temperature for imine in comparison with its mixture with PTB7 was observed for di(FPh-ThAz-An)-TPA, with an almost double difference in temperature. In only one case, the presence of PTB7 increased the thermal response—namely for di(FPh-ThAz-An)-TPA—with a difference of maximal temperatures of 38 °C.

## 4. Conclusions 

We have synthesized four new imines tuned by concentration and presence of PTB7, and investigated their UV–Vis absorption properties with the purpose of finding the optimal concentration in the composition with PTB7 for organic photovoltaics. The wavelength of the absorption peak of imines was not shifted with increasing concentration or PTB7 addition. All PTB7:imine compositions exhibited wide absorptions of up to 800 nm and isosbestic points in the range of 440–540 nm, confirming changes in the macromolecular organization of investigated compounds.

All of the investigated imines fulfilled a weak absorption range in the UV–Vis spectrum of PTB7—from 350 to 550 nm—and could be used as components of active layers in polymer solar cells. However, the best candidate was di-BenzThAz-terTh, for which the maxima of the absorption lay at approximately 450 nm, which is the middle of weak absorption range of PTB7.

In summary, we can conclude that the difference in the observed IR thermal behavior of imines and their mixture with PTB7 can be related to the specific organization of molecules in the organic layer. Considering the molecular structure of studied imines, only two of them, i.e., 3,5-di(diMeTPA)-1,2,4-ThdiAz and di-BenzThAz-terTh—whose structures are symmetrical—can adapt a linear-like form. For these two compounds, the behaviors of pure imines and their mixtures with PTB7 are almost equal, which may be due to slight interference caused by PTB7 in the created layers. On the other hand, in the case of the other two imines, i.e., di (FPh-ThAz-An) -TPA and 2-diMeTPA-ThdiAz, we deal with compounds whose structures are not symmetrical or have a certain angularity of substituents. These two factors cause differences in the behaviors of layers containing only imines and their mixtures with PTB7. Thus, the mixed layer has a different behavior, significantly different from pure imine.

## Figures and Tables

**Table 1:** Selected UV–Vis properties of PTB7, 3,5-di(diMeTPA)-1,2,4-ThdiAz, and PTB7: 3,5-di(diMeTPA)-1,2,4-ThdiAz.

Code	C(M)	λ_max_(nm)	A	ε(M^−1^cm^−1^)
PTB7	2.48 × 10^−5^	680 621 300	0.4136 0.3210 0.1868	166,774 129,435 75,323
3,5-di(diMeTPA)-1,2,4-ThdiAz	1.01 × 10^−5^	300 375	0.2654 0.2616	26,277 25,901
	2.03 × 10^−5^	300 375	0.5952 0.5686	29,320 28,010
	3.04 × 10^−5^	300 375	0.9212 0.8719	30,303 28,681
	4.07 × 10^−5^	300 375	1.2283 1.1790	30,179 28,968
	5.07 × 10^−5^	300 375	1.5581 1.4330	30,732 28,264
PTB7: 3,5-di(diMeTPA)-1,2,4-ThdiAz	1.01 × 10^−5^	300 370 620 680	0.5083 0.3375 0.2638 0.3256	50,327 33,416 26,119 32,238
	2.03 × 10^−5^	300 370 620 680	0.8083 0.6250 0.2796 0.3306	39,818 30,788 13,773 16,286
	3.04 × 10^−5^	300 370 620 680	1.1167 0.9417 0.2796 0.3364	36,734 30,977 9197 11,066
	4.07 × 10^−5^	300 370 620 680	1.4583 1.2667 0.2884 0.3404	35,830 31,123 7086 8364
	5.07 × 10^−5^	300 370 620 680	1.7875 1.5833 0.2913 0.3453	35,256 31,229 5746 6811

**Table 2:** Selected UV–Vis properties of 2-diMeTPA-1,3,4-ThdiAz and PTB7: 2-diMeTPA-1,3,4-ThdiAz.

Code	C (M)	λ_max_ (nm)	A	ε (M^−1^cm^−1^)
2-diMeTPA-1,3,4-ThdiAz	4.30 × 10^−5^	301 400	0.5341 0.2640	12,421 6140
	8.60 × 10^−5^	301 400	1.1542 0.5526	13,421 6426
	1.29 × 10^−4^	301 400	1.7497 0.8472	5812 6777
	1.72 × 10^−4^	301 400	2.3637 1.1419	13,742 6639
	2.15 × 10^−4^	301 400	2.9100 1.4243	13,535 6625
PTB7: 2-diMeTPA-1,3,4-ThdiAz	4.30 × 10^−5^	300 395 620 680	0.7655 0.3410 0.3240 0.3924	17,802 7930 7535 9126
	8.60 × 10^−5^	300 395 620 680	1.3445 0.6240 0.3474 0.4060	15,634 7256 4040 4721
	1.29 × 10^−4^	300 395 620 680	1.9557 0.9328 0.3597 0.4161	15,160 7231 2788 3226
	1.72 × 10^−4^	300 395 620 680	2.5214 1.1929 0.3802 0.4311	14,659 6935 2210 2506
	2.15 × 10^−4^	300 395 620 680	3.1179 1.5183 0.3921 0.4378	14,502 7062 1824 2036

**Table 3:** Selected UV–Vis properties of di(FPh-ThAz-An)-TPA and PTB7: di(FPh-ThAz-An)-TPA.

Code	C (M)	λ_max_ (nm)	A	ε (M^−1^cm^−1^)
di(FPh-ThAz-An)-TPA	1.06 × 10^−5^	295 333 385	0.2170 0.3309 0.2116	20,472 31,217 19,962
	2.11 × 10^−5^	295 333 385	0.4882 0.6726 0.4340	23,137 31,877 20,569
	3.17 × 10^−5^	295 333 385	0.8245 1.0415 0.6781	26,009 32,855 21,391
	4.22 × 10^−5^	295 333 385	1.1825 1.4538 0.9167	28,021 34,450 21,723
	5.28 × 10^−5^	295 333 385	1.5514 1.8986 1.1771	29,383 35,958 22,294
PTB7:di(FPh-ThAz-An)-TPA	1.06 × 10^−5^	295 332 385 621 683	0.5153 0.4936 0.3255 0.4014 0.5262	48,613 46,566 30,707 37,868 49,641
	2.11 × 10^−5^	295 332 385 621 683	0.7703 0.8245 0.3479 0.4014 0.5262	36,507 39,076 16,488 19,024 24,938
	3.17 × 10^−5^	295 332 385 621 683	1.0795 1.1880 0.7703 0.4014 0.5262	34,054 37,476 24,300 12,662 16,599
	4.22 × 10^−5^	295 332 385 621 683	1.4375 1.6002 1.0252 0.4014 0.5262	34,064 37,919 24,294 9512 12,469
	5.28 × 10^−5^	295 332 385 621 683	1.8769 2.1590 1.3019 0.4014 0.5262	35,547 40,890 24,657 7602 9966

**Table 4:** Selected UV–Vis properties of di-BenzThAz-terTh and PTB7: di-BenzThAz-terTh.

Code	C (M)	λ_max_ (nm)	A	ε (M^−1^cm^−1^)
di-BenzThAz-terTh	1.60 × 10^−5^	457	0.8102	50,638
	3.19 × 10^−5^	457	1.7033	53,395
	4.79 × 10^−5^	457	2.5002	52,196
	6.37 × 10^−5^	457	3.3795	53,053
	7.97 × 10^−5^	457	4.2177	52,920
PTB7:di-BenzThAz-terTh	1.60 × 10^−5^	458 620 680	0.9371 0.2978 0.3423	58,569 18,613 21,394
	3.19 × 10^−5^	458 620 680	1.7815 0.2813 0.3423	55,846 8818 10,730
	4.79 × 10^−5^	458 620 680	2.5950 0.2997 0.3423	54,175 6257 7146
	6.37 × 10^−5^	458 620 680	3.5526 0.2992 0.3578	55,771 4697 5617
	7.97 × 10^−5^	458 620 680	4.3455 0.2986 0.3578	54,523 3746 4489

## Supplementary Materials

The following are available online at https://www.mdpi.com/1996-1944/12/24/4191/s1, Figure S1: Theoretical IR spectra of 3,5-di(diMeTPA)-1,2,4-ThdiAz along with theoretical vibrational frequencies. Figure S2: Theoretical IR spectra of 2-diMeTPA-1,3,4-ThdiAz along with theoretical vibrational frequencies. Figure S3: Theoretical IR spectra of di-BenzThAz-terTh along with theoretical vibrational frequencies. Figure S4: Theoretical IR spectra of di(FPh-ThAz-An)-TPA along with theoretical vibrational frequencies. Table S1: Theoretical calculations of LUMO-HOMO levels of imines.

## References

[1] Iwan A., Sek D. (2008). Processible polyazomethines and polyketanils: From aerospace to light emitting diodes and other advanced applications. Prog. Polym. Sci..

[2] Sek D., Iwan A., Jarzabek B., Kaczmarczyk B., Kasperczyk J., Mazurak Z., Domanski M., Karon K., Lapkowski M. (2008). Hole transport triphenylamine-azomethine conjugated system: Synthesis and optical, photoluminescence and electrochemical properties. Macromolecules.

[3] Iwan A., Sek D. (2011). Polymers with Triphenylamine Units: Photonic and Electroactive Materials. Prog. Polym. Sci..

[4] Bolduc A., Mallet C., Skene W.G. (2012). Survey of recent advances of in the field of π-conjugated heterocyclic azomethines as materials with tuneable properties. Sci. China Chem..

[5] Işık D., Santato C., Barik S., Skene W.G. (2012). Charge-Carrier Transport in Thin Films of π-Conjugated Thiopheno-Azomethines. Org. Electron..

[6] Tshibaka T., Bishop S., Dufresne S., Roche Ulliel I., Lubell W.D., Skene W.G. (2011). Conjugated 4-methoxybipyrrole thiophene azomethines: Synthesis, opto-electronic properties and crystallographic characterization. Chem. Eur. J..

[7] Minkin V.I., Tsukanov A.V., Dubonosov A.D., Bren V.A. (2011). Tautomeric Schiff bases: Iono-, solvato-, thermo- and photochromism. J. Mol. Struct..

[8] Bourque A.N., Dufresne S., Skene W.G. (2009). Thiophene-Phenyl Azomethines with Varying Rotational Barriers—Model Compounds for Examining Imine Fluorescence Deactivation. J. Phys. Chem. C.

[9] Barik S., Bletzacker T., Skene W.G. (2012). π-Conjugated Fluorescent Azomethine Copolymers: Opto-Electronic, Halochromic, and Doping Properties. Macromolecules.

[10] Niu H., Luo P., Zhang M., Zhang L., Hao L., Luo J., Bai X., Wang W. (2009). Multifunctional, photochromic, acidichromic, electrochromic molecular switch: Novel aromatic poly(azomehine)s containing triphenylamine group. Eur. Polym. J..

[11] Lu L., Yu L. (2014). Understanding low bandgap polymer PTB7 and optimizing polymer solar cells based on it. Adv. Mater..

[12] He Z., Zhong C., Su S., Xu M., Wu H., Cao Y. (2012). Enhanced power-conversion efficiency in polymer solar cells using an inverted device structure. Nat. Photonics.

[13] Liu C., Wang K., Hu X., Yang Y., Hsu C.-H., Zhang W., Xiao S., Gong X., Ca Y. (2013). On the role of molecular weight and homocoupling defects in organic solar cells. ACS Appl. Mat. Interfaces.

[14] Du X., Heumueller T., Gruber W., Classen A., Unruh T., Li N., Brabec C.J. (2019). Efficient Polymer Solar Cells Basedon Non-fullerene Acceptors with Potential Device Lifetime Approaching 10 Years. Joule.

[15] Hou J., Inganas O., Friend R.H., Gao F. (2018). Organic solar cells based on non-fullerene acceptors. Nat. Mater..

[16] Cheng P., Li G., Zhan X., Yang Y. (2018). Next-generation organic photovoltaics based on non-fullerene acceptors. Nat. Photonics.

[17] Li S., Ye L., Zhao W., Zhang S., Mukherjee S., Ade H., Hou J. (2016). Energy-level modulation of small-molecule electron acceptors to achieve over 12% efficiency in polymer solar cells. Adv. Mater..

[18] Gaspar H., Figueira F., Pereira L., Mendes A., Viana J.C., Bernardo G. (2018). Recent Developments in the Optimization of the Bulk Heterojunction Morphology of Polymer: Fullerene Solar Cells. Materials.

[19] Savikhin V., Jagadamma L.K., Purvis L.J., Robertson I., Oosterhout S.D., Douglas C.J., Samuel I.D.W., Toney M.F. (2018). Morphological, Chemical, and Electronic Changes of the Conjugated Polymer PTB7 with Thermal Annealing. IScience.

[20] Roehling J.D., Baran D., Sit J., Kassar T., Ameri T., Unruh T., Brabec C.J., Moulé A.J. (2016). Nanoscale Morphology of PTB7 Based Organic Photovoltaics as a Function of Fullerene Size. Sci. Rep..

[21] Park S., Jeong J., Hyun G., Kim M., Lee H., Yi Y. (2016). The origin of high PCE in PTB7 based photovoltaics: Proper charge neutrality level and free energy of charge separation at PTB7/PC71BM interface. Sci. Rep..

[22] Petrus M.L., Bouwer R.K.M., Lafont U., Athanasopoulos S., Greenham N.C., Dingemans T.J. (2014). Small-molecule azomethines: Organic photovoltaics via Schiff base condensation chemistry. J. Mater. Chem. A.

[23] Canli N.Y., Safak-Boroglu M., Bilgin-Eran B., Gunes S. (2014). Bilayer polymer/fullerene solar cells with a liquid crystal. Thin Solid Film..

[24] Moussalem C., Segut O., Gohier F., Allain M., Frere P. (2014). Facile Access via Green Procedures to a Material with the Benzodifuran Moiety for Organic Photovoltaics. ACS Sustain. Chem. Eng..

[25] Jeevadason A.W., Murugavel K.K., Neelakantan M.A. (2014). Review on Schiff bases and their metal complexes as organic photovoltaic materials. Renew. Sustain. Energy Rev..

[26] Tan Q., Zhang X., Mao L., Xin G., Zhang S. (2013). Novel zinc porphyrin sensitizers for dye-sensitized solar cells: Synthesis and spectral, electrochemical, and photovoltaic properties. J. Mol. Struct..

[27] Petrus M.L., Morgenstern F.S.F., Sadhanala A., Friend R.H., Greenham N.C., Dingemans T.J. (2015). Device Performance of Small-Molecule Azomethine-Based Bulk Heterojunction Solar Cells. Chem. Mater..

[28] Petrus M.L., Bein T., Dingemans T.J., Docampo P. (2015). A low cost azomethine-based hole transporting material for perovskite photovoltaics. J. Mater. Chem. A.

[29] Bogdanowicz K.A., Iwan A. (2019). Symmetrical Imines and the Method of Obtaining Thereof. PL Patent.

[30] Bogdanowicz K.A., Iwan A. (2019). Symmetrical Imines with Terthiophene Core and the Method of Obtaining Thereof. PL Patent.

[31] Bogdanowicz K.A., Iwan A. (2019). Symmetrical Imines with Trirphenylamine Core and the Method of Obtaining Thereof. PL Patent.

[32] Frisch M.J., Trucks G.W., Schlegel H.B., Scuseria G.E., Robb M.A., Cheeseman J.R., Scalmani G., Barone V., Petersson G.A., Nakatsuji H. (2016). Gaussian 16, Revision A.03.

[33] Kohn W., Sham L.J. (1965). Self-Consistent Equations Including Exchange and Correlation Effects. Phys. Rev..

[34] Becke A.D. (1988). Density-functional exchange-energy approximation with correct asymptotic behavior. Phys. Rev. A.

[35] Lee C., Yang W., Parr R.G. (1988). Development of the Colle-Salvetti correlation-energy formula into a functional of the electron density. Phys. Rev. B.

[36] Różycka A., Bogdanowicz K.A., Górska N., Rysz J., Marzec M., Iwan A., Pich R., Januszko A. (2019). Influence of TiO_2_ nanoparticles on liquid crystalline, structural and electrochemical properties of (8z)-n-(4-((z)-(4-pentylphenylimino)methyl)benzylidene)-4-pentylbenzenamine. Materials.

[37] Korona K.P., Korona T., Rutkowska-Zbik D., Grankowska-Ciechanowicz S., Iwan A., Kamińska M. (2015). Polyazomethine as a component of solar cells—Theoretical and optical study. J. Phys. Chem. Solids.

