# Supplementary material for: UV–Vis Absorption Properties of New Aromatic Imines and Their Compositions with Poly({4,8-bis[(2-Ethylhexyl)oxy]Benzo[1,2-b:4,5-b′]Dithiophene-2,6-diyl}{3-Fluoro-2-[(2-Ethylhexyl)Carbonyl]Thieno[3,4-b]Thiophenediyl})
Source: Materials (Basel). 2019 Dec 13;12(24):4191. doi: 10.3390/ma12244191 (PMC6947379; doi:10.3390/ma12244191)
Supplement: Supplementary file 1 [file materials-12-04191-s001.pdf]

materials

Article

UV–Vis Absorption Properties of New Aromatic Imines and Their Compositions with Poly({4,8-bis[(2-Ethylhexyl)oxy]Benzo[1,2-b:4,5-b′]Dithiophene-2,6-diyl}{3-Fluoro-2-[(2-Ethylhexyl)Carbonyl]Thieno[3,4-b]Thiophenediyl})

Agnieszka Gonciarz [1], Robert Pich [1], Krzysztof Artur Bogdanowicz [2], Beata Jewloszewicz [2], Wojciech Przybył [2], Karolina Dysz [2], Agnieszka Dylong [2], Anna Kwak [2], Andrzej Kaim [3], Agnieszka Iwan [2,*], Jaroslaw Rusin [1] and Adam Januszko [2]

1 General Tadeusz Kosciuszko Military University of Land Forces, Wroclaw; MULF Wroclaw, Faculty of Security and Safety Research, Czajkowskiego 109 Str., 51–147 Wroclaw, Poland; agnieszka.gonciarz@awl.edu.pl (A.G.); robert.pich@awl.edu.pl (R.P); jaroslaw.rusin@awl.edu.pl (J.R.)
2 Military Institute of Engineer Technology, Obornicka 136 Str., 50–961 Wroclaw, Poland; bogdanowicz@witi.wroc.pl (K.A.B.); jewloszewicz@witi.wroc.pl (B.J.); przybyl@witi.wroc.pl (W.P.); dysz@witi.wroc.pl (K.D.); dylong@witi.wroc.pl (A.D.); kwak@witi.wroc.pl (Anna Kwak); januszko@witi.wroc.pl (A.J.)
3 Faculty of Chemistry, University of Warsaw, Pasteura 1, 02–093 Warsaw, Poland; akaim@chem.uw.edu.pl
* Correspondence: iwan@witi.wroc.pl

Received: 7 November 2019; Accepted: 9 December 2019; Published: date

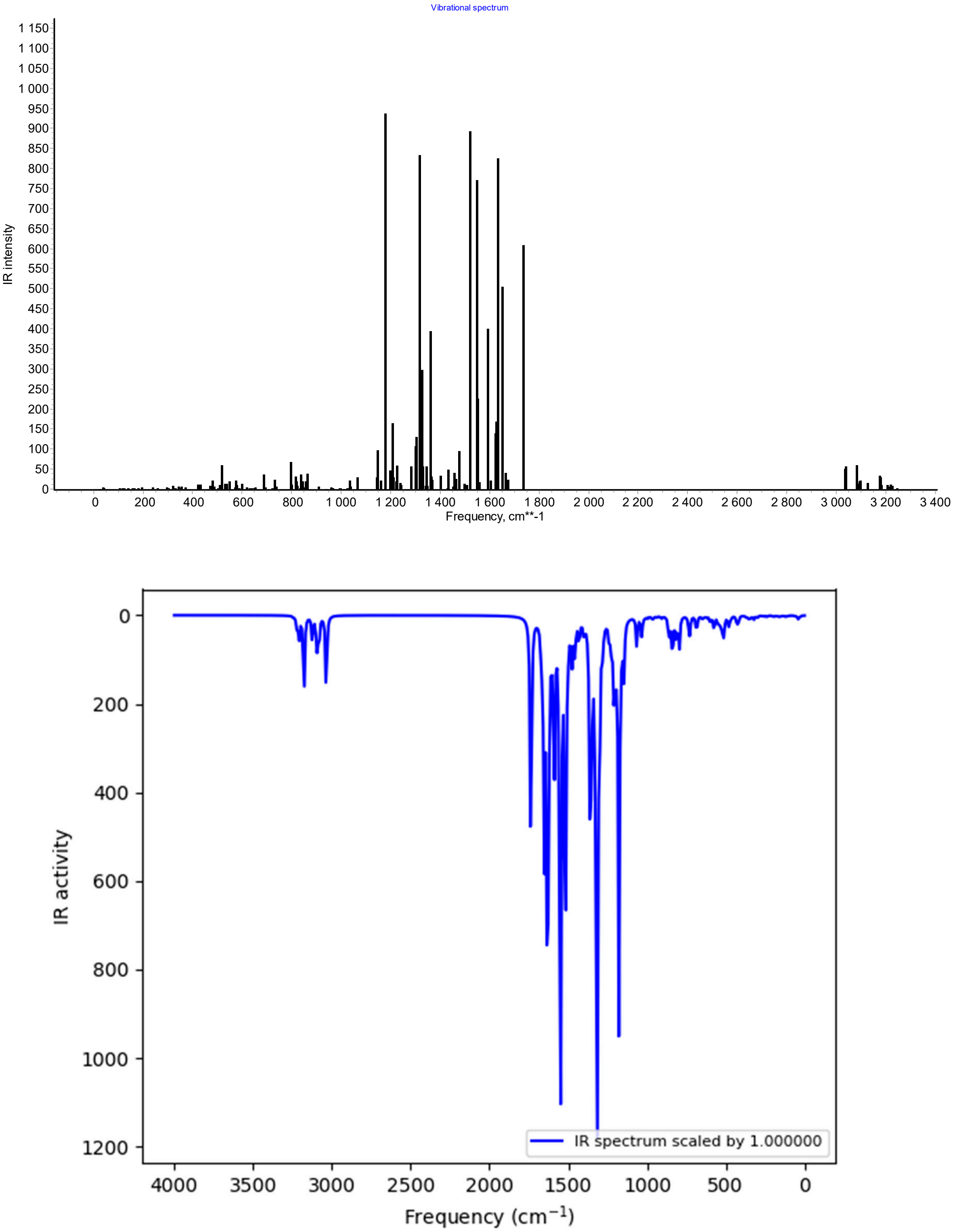

Figure S1. Theoretical IR spectra of 3,5-di(diMeTPA)-1,2,4-ThdiAz along with theoretical vibrational frequencies.

Frequency,cm^{-1}	Intensity
3.5664	0.1269
3.6645	0.2697

4.2903	0.0374
18.0062	0.0114
21.1521	0.1588
25.0397	0.3179
27.1289	0.4475
27.7658	0.666
28.74	0.3631
29.7064	0.1989
31.2567	0.0904
37.6595	1.0896
38.352	4.2397
40.2853	2.0507
42.8224	1.6619
44.713	0.4874
49.2794	0.3838
56.9741	0.5768
60.3724	0.6969
66.549	0.3197
79.5577	0.3582
85.2462	0.1914
104.2932	1.2293
115.9937	1.4584
122.6182	2.5545
139.757	1.107
149.0603	0.8094
156.776	2.2876
164.4558	2.1847
179.2366	1.2114
195.1956	3.3343
206.1418	0.6869
227.0163	0.1751
238.1034	3.4975
257.502	1.0424
258.8559	1.1526
272.0208	0.7585
296.0799	2.9718
296.8076	0.4846
302.5587	2.0834
319.8653	7.9047
323.6646	1.1543
330.9633	1.8032
342.7096	5.531

353.0229	2.7147
355.5563	4.8464
361.2105	0.2766
366.3372	0.2922
370.0337	3.4342
382.2404	0.4818
390.4031	0.2728
419.8744	11.4258
422.6219	2.6282
422.9882	2.6988
424.3395	2.9506
426.5833	0.7926
430.6798	12.5485
460.7358	0.5234
469.1947	6.7724
479.4415	21.3031
486.2049	6.3718
501.8504	1.2517
508.6678	10.6223
516.3709	59.7386
529.2976	13.4959
534.8637	12.9422
548.4973	20.0569
571.8239	3.8197
573.6364	21.6179
575.6738	9.5213
586.7596	2.2401
598.7467	14.0365
618.0002	3.3858
630.3517	1.9516
639.9658	2.7327
647.7247	1.7397
650.9492	3.2205
652.4295	0.8854
653.233	0.5786
658.4268	0.171
658.585	0.0494
685.1311	37.043
691.5125	4.4947
722.3252	2.8048
723.5014	2.0967
729.6933	15.3904

730.0474	23.4089
735.6361	6.5133
737.1724	5.9059
782.7815	0.3817
794.8651	67.4406
797.7176	3.2329
798.4387	11.1775
813.609	31.5174
817.3695	18.5777
821.6955	8.644
831.0968	0.6125
834.1403	1.8293
836.5575	35.6276
837.6823	36.5643
839.111	1.6314
839.7735	3.655
841.6663	19.3092
845.9375	2.9106
848.1719	1.894
849.0381	0.8527
849.4582	0.131
856.4935	18.7886
861.9248	38.3715
907.823	5.3291
935.5633	0.7689
937.704	0.2015
938.7953	0.8791
938.9601	0.6562
958.9545	3.0423
962.8324	1.5949
963.2011	0.5969
964.7922	0.1352
965.0196	0.4762
966.0553	1.19
966.8976	0.7277
968.4903	0.1365
968.5875	0.836
969.5656	0.8185
969.5743	0.1684
992.3552	1.9982
996.5666	2.4435
1011.8068	0.4186

1012.157	0.4519
1012.566	0.4763
1012.8504	0.7884
1024.9607	2.1221
1027.1542	2.3874
1032.7181	10.395
1034.6379	20.2979
1035.7801	6.5124
1035.8856	1.6509
1036.4447	2.3143
1036.7416	4.8169
1064.6713	12.1509
1064.8795	14.5653
1064.9807	28.8598
1065.0451	12.6335
1144.6254	28.0815
1145.4275	10.6851
1146.5345	1.3108
1146.9397	1.1264
1148.1386	97.4762
1160.089	21.1825
1177.9104	937.0811
1196.1265	46.6176
1198.3272	21.2996
1207.6193	165.2157
1207.7648	2.2355
1208.1352	2.0446
1208.5038	28.5389
1221.5358	18.8696
1223.5418	58.2362
1238.5279	7.3753
1238.6825	7.1333
1238.9145	15.1063
1238.9492	12.444
1239.567	9.7852
1280.8626	55.6231
1298.8493	106.0964
1302.4934	130.1001
1315.2758	833.2981
1317.162	69.5841
1317.9509	273.287
1324.5417	298.5715

1327.7823	57.0054
1342.2445	8.2064
1342.4824	1.4464
1344.8952	21.4493
1345.1371	56.9018
1345.3779	22.2327
1348.0126	7.6469
1358.8381	394.3517
1359.2659	202.2593
1364.0159	31.167
1367.2089	23.5955
1399.9628	33.7166
1427.2611	1.4974
1427.491	1.1769
1427.4965	1.0921
1427.6973	2.0327
1431.7924	48.7035
1450.3793	5.8911
1451.1164	3.6252
1457.4765	40.7612
1458.4696	14.5816
1462.3761	24.2926
1476.4906	95.3624
1497.4171	14.3934
1501.6375	4.7682
1501.6656	5.273
1501.7556	4.3488
1501.8143	4.701
1506.4628	8.1025
1506.9156	10.3629
1507.6157	3.7066
1507.9962	1.0806
1518.3672	893.5263
1548.2395	772.194
1550.6216	178.497
1551.5669	151.3371
1551.8199	227.0187
1555.942	18.3252
1556.5908	4.7767
1589.6956	400.2502
1603.3422	22.1753
1621.3736	139.5092

1622.7211	11.1651
1627.0661	168.3875
1627.3161	19.8993
1632.368	825.7135
1651.9708	505.0783
1663.5294	15.1944
1663.7993	40.6751
1663.92	10.1163
1671.4394	22.5073
1673.2893	23.1053
1736.7642	608.1787
3034.7849	44.2766
3035.1959	49.9128
3036.2439	55.9885
3036.3951	35.3338
3081.3764	59.1527
3084.7395	13.9941
3095.0306	20.09
3095.2657	19.587
3096.5599	20.6283
3096.6895	18.9386
3123.6479	16.376
3123.6578	13.0857
3124.695	14.4291
3125.0215	14.241
3175.426	14.4625
3175.5253	19.1254
3176.2063	33.0313
3176.4575	18.0516
3176.7837	17.5129
3176.8565	13.2553
3176.9425	16.3715
3177.5295	28.487
3177.8073	29.223
3180.6186	9.1794
3207.0789	9.4486
3207.1817	9.1761
3207.3362	10.6207
3207.4122	8.5788
3208.2547	1.9971
3208.3499	2.1506
3208.6783	2.9595

3208.6894	3.609
3210.2423	2.8436
3216.0596	5.1501
3218.6915	11.0762
3222.1002	8.5829
3224.2742	7.7429
3244.6555	2.1724

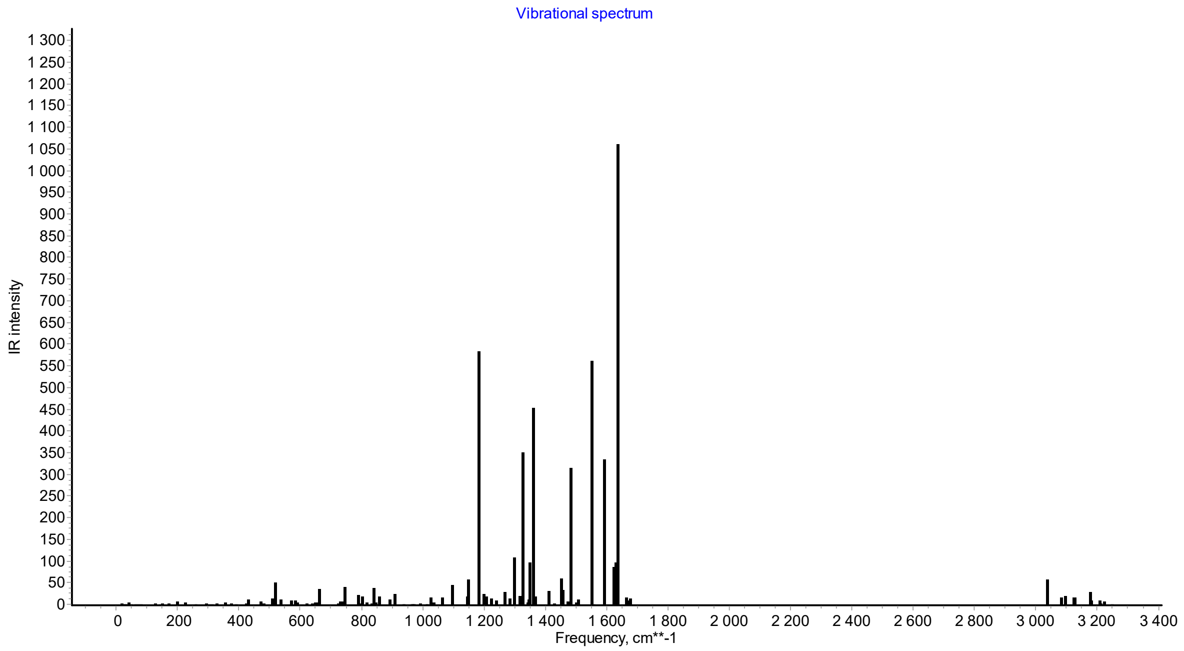

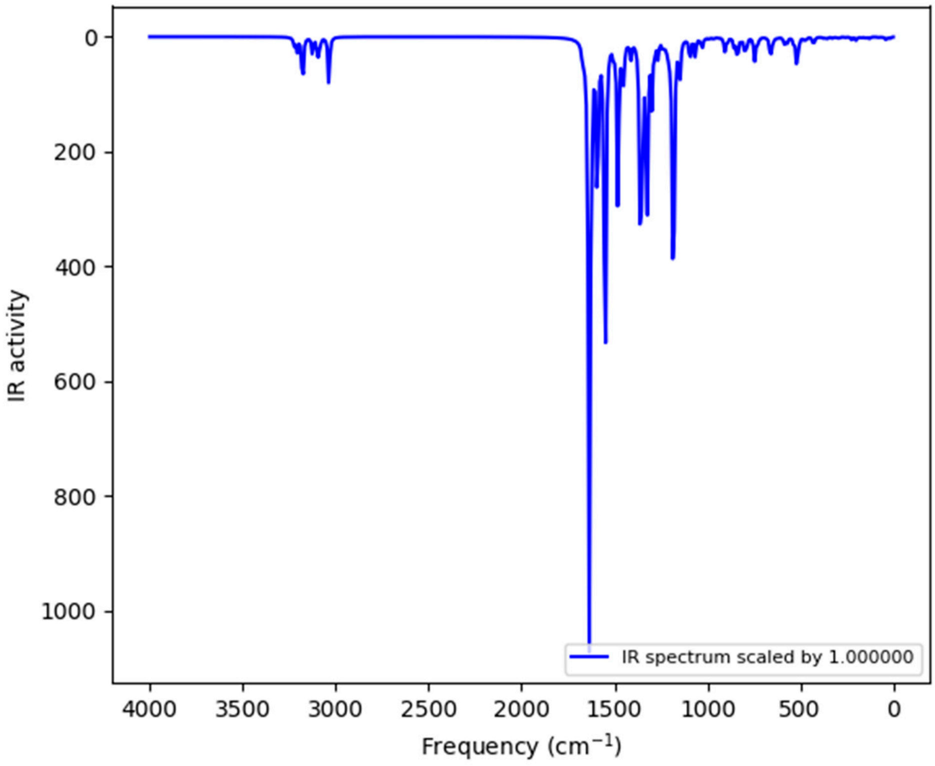

Figure S2. Theoretical IR spectra of 2-diMeTPA-1,3,4-ThdiAz along with theoretical vibrational frequencies.

Frequency, cm^{-1},	Intensity
17.3199	2.6025
20.6411	0.2263
26.1015	0.2578
28.6188	0.3462
33.0159	0.1593
38.5848	4.0921
40.8007	0.8803
49.3396	0.6074
62.009	0.2268
72.241	0.2421
80.527	0.9478
127.2379	3.058
143.2223	0.7908
149.5028	1.8055
171.1131	1.621
198.7696	6.633
224.227	4.8935
257.5338	1.0718
292.079	2.6588
301.2705	0.9497
312.5097	0.2415
327.0521	2.5367
354.7467	0.2016
356.1389	3.8008
373.5824	2.7442
390.5568	0.2568
423.0256	2.5593
424.2724	2.6709
430.3502	10.3725
469.8704	5.6312
480.9668	3.1632
509.4025	13.2758
518.7869	51.6527
534.7072	11.2505
570.5888	9.8447
582.9516	9.4384
588.4794	4.8469
621.7732	1.6279

639.1667	1.6107
647.1024	5.1884
651.7857	3.4723
658.3309	3.8884
661.1032	35.6289
723.5957	2.0851
728.941	7.0946
736.4606	5.7987
746.194	40.3914
789.9851	22.4103
797.8039	2.4328
800.9518	17.1993
818.0925	3.9975
833.8097	1.7814
837.7266	36.5814
839.8372	2.581
845.9162	3.3393
849.5253	0.1424
856.0918	17.164
893.3542	10.2436
906.8021	24.6318
935.6696	0.2711
938.9534	0.6226
962.8947	0.5428
965.0566	0.6835
966.3037	0.411
968.6978	0.6122
969.6642	0.6233
991.1589	2.8405
1012.2867	0.4176
1012.9944	0.5756
1024.9359	1.8763
1027.4001	14.5995
1035.899	2.8574
1036.7593	3.3465
1064.9365	16.2778
1065.0574	16.4114
1093.6344	44.5354
1144.7063	17.3277
1146.5289	1.0486
1148.8627	56.6153
1182.6883	583.8966

1198.2574	24.8615
1207.7958	0.6108
1208.4365	17.2967
1221.5735	13.3378
1238.5994	6.9955
1238.9382	8.9601
1265.7973	29.6554
1283.598	14.0717
1298.6071	107.2365
1317.4636	19.0316
1325.1258	350.319
1342.3436	3.9756
1344.9279	10.4117
1346.946	97.3814
1359.0838	454.1718
1367.3005	18.0634
1411.4783	30.0704
1427.5405	1.0543
1427.7507	1.6322
1449.5008	58.6411
1451.0923	3.4533
1457.3325	33.1424
1473.8599	6.4967
1481.2643	314.5199
1501.6966	5.381
1501.7985	5.2072
1506.4609	10.3626
1507.6366	0.583
1549.8349	561.2458
1551.574	162.2362
1556.3665	0.7835
1591.8395	335.0753
1621.9217	86.726
1627.2931	97.3364
1636.1781	1059.8036
1663.5062	14.5748
1665.6115	8.5093
1674.3677	14.2898
3036.3913	56.144
3036.5226	31.6922
3084.0338	15.3705
3096.7586	20.5138

3096.8822	19.0712
3124.9549	14.3315
3125.2291	14.333
3177.0831	11.6109
3177.1399	18.1359
3177.694	27.7125
3177.9902	27.2272
3180.7078	9.5499
3206.9413	9.4411
3207.0202	8.3493
3208.3444	1.7317
3208.4738	4.2678
3209.8075	2.2845
3222.7377	7.5221
3224.1815	6.729
3249.088	0.658

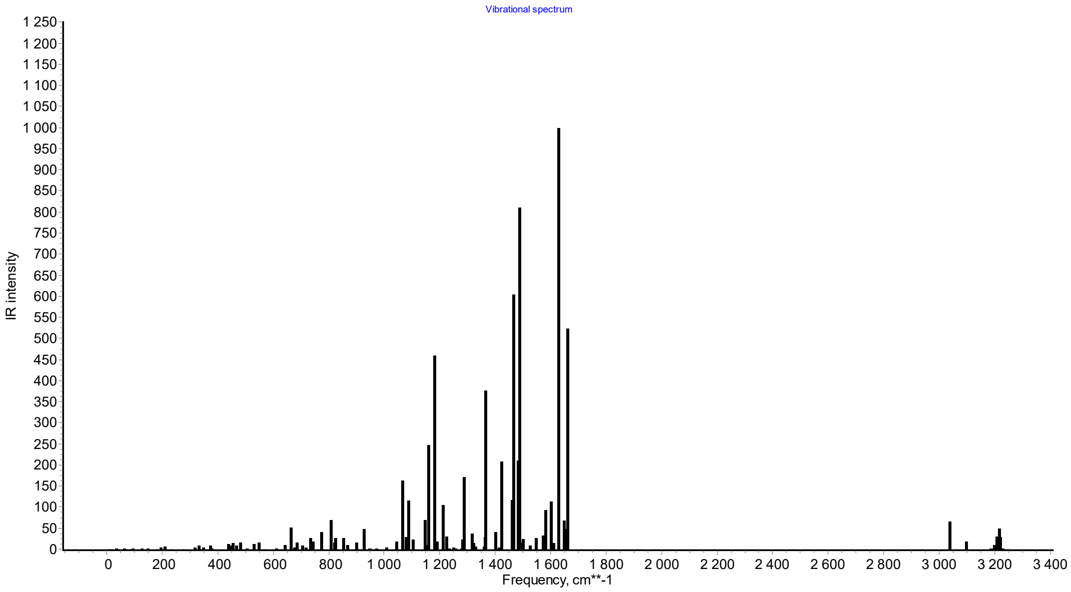

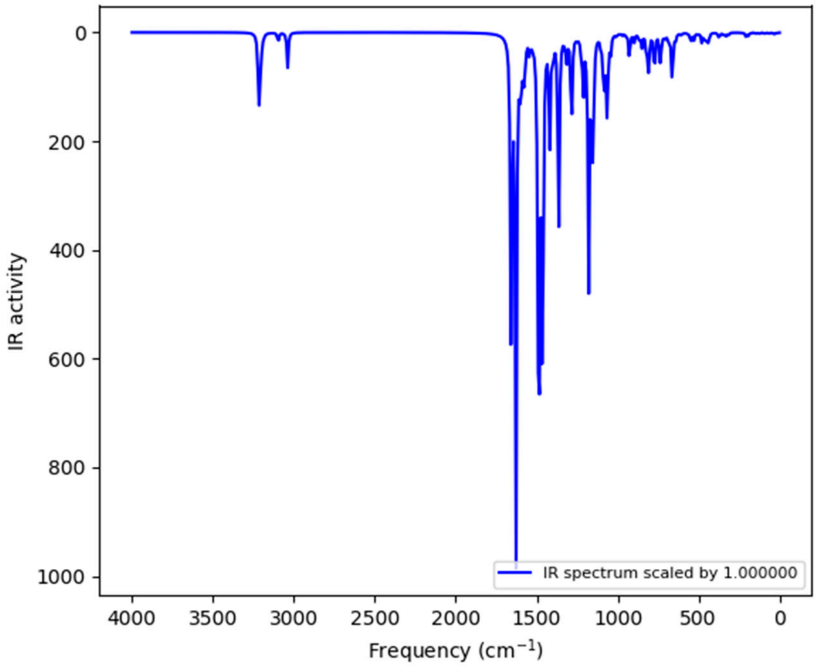

Figure 3. Theoretical IR spectra of di-BenzThAz-terTh along with theoretical vibrational frequencies.

Frequency,cm^{-1}	Intensity
4.4043	0.0502
4.7982	0.3143
10.2748	0.1564
12.7428	0.1052
14.1543	0.0336
21.9735	0.7142
30.3782	0.0641
33.9568	2.9899
44.4596	0.5157
62.4308	1.2088
67.9017	0.93
84.8039	0.315
92.8128	2.2841
124.2929	2.6824
138.6159	0.0089
147.025	2.1547
177.3571	0.8157
192.0172	4.7526
197.3397	0.2928
198.4565	0.8766
199.3743	0.8119
208.7411	6.7909

230.3524	0.3956
254.7904	0.0656
274.9794	0.151
315.0853	4.5602
331.242	7.4032
343.8048	0.8711
347.2301	3.2331
361.6474	0.3157
373.0801	9.0594
379.1547	2.3231
381.2678	0.181
437.4424	12.7839
443.9774	7.7222
445.2496	3.7537
453.423	13.51
465.9535	8.2801
480.444	17.4595
503.7422	2.7613
511.8689	0.0927
520.5796	0.4647
529.5462	13.0302
546.0288	2.8611
548.567	15.833
558.2534	0.0992
567.1734	0.967
576.9905	0.0418
602.8918	0.7062
608.2007	0.0333
608.8098	3.0376
630.3597	0.4819
642.5388	10.8963
643.6218	0.2699
661.9908	51.8049
663.4707	48.3332
674.5136	4.5164
685.0713	17.2331
704.0121	7.539
715.8048	3.4059
716.9615	1.1619
732.9825	26.9815
736.9668	10.8222
738.195	3.2166

738.7045	10.1626
740.2795	18.4116
772.5168	40.3829
774.0743	40.963
808.1773	69.8073
811.2461	0.0533
819.5985	16.2154
822.732	26.324
850.88	26.3381
868.2307	11.0785
868.8462	0.5134
870.4023	0.4574
897.333	15.7998
901.6706	0.0066
906.3566	0.0797
907.7691	0.2712
927.1987	1.7619
927.4005	48.2247
946.5303	1.5501
948.252	1.9912
970.0777	3.0552
986.1074	0.0004
987.1206	0.0022
1007.9902	4.1152
1043.4896	15.7763
1043.7495	18.8719
1064.1282	162.2884
1076.431	28.6264
1080.4801	0.7765
1085.7361	116.1221
1102.6772	22.7299
1146.3254	69.7581
1150.6025	9.6876
1157.0092	246.8681
1178.7254	459.608
1188.8751	2.2163
1190.4781	18.2444
1211.9126	106.0737
1223.6661	31.6594
1233.4794	2.5394
1247.7828	3.7422
1255.1058	2.9865

1277.0777	2.3668
1278.3941	1.4731
1281.3109	21.6755
1285.8312	171.1406
1314.3838	37.1368
1319.741	14.6906
1327.7733	6.0094
1358.7013	7.1569
1361.5906	27.8385
1364.8933	377.4349
1399.2002	40.4017
1411.8278	4.8456
1420.2934	208.0614
1458.5418	117.677
1464.6572	604.6343
1480.3582	209.9522
1487.5	809.6394
1497.348	14.2157
1498.2134	5.5132
1499.3455	25.3602
1526.1496	8.0398
1545.3445	27.517
1570.1143	33.2472
1582.184	92.7869
1599.289	113.2079
1606.5421	3.6445
1608.791	14.4478
1628.2716	998.9212
1648.1004	68.0055
1651.1354	46.6936
1659.1026	522.6653
3038.4317	65.0831
3097.1409	18.2705
3185.9383	2.3791
3186.618	2.5596
3197.3799	7.8118
3198.8498	10.1975
3208.2252	30.2735
3209.3528	30.8348
3215.7749	49.1655
3215.9565	12.4832
3216.7021	26.7014

3217.9919	29.4543
3220.2329	14.1757
3230.1254	1.7885
3230.6513	0.1675
3248.1745	0.3993

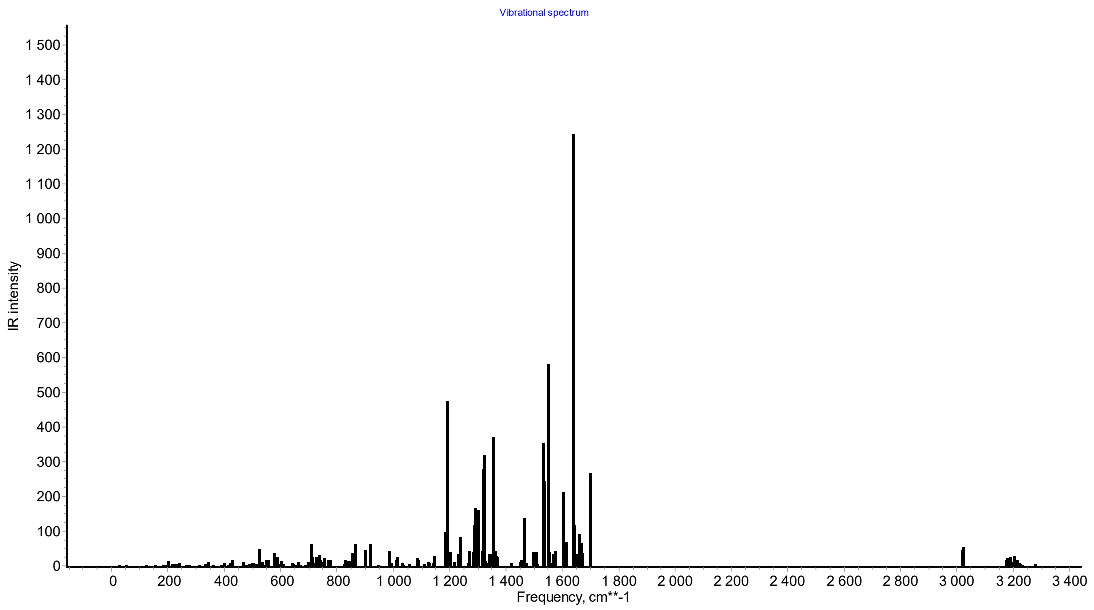

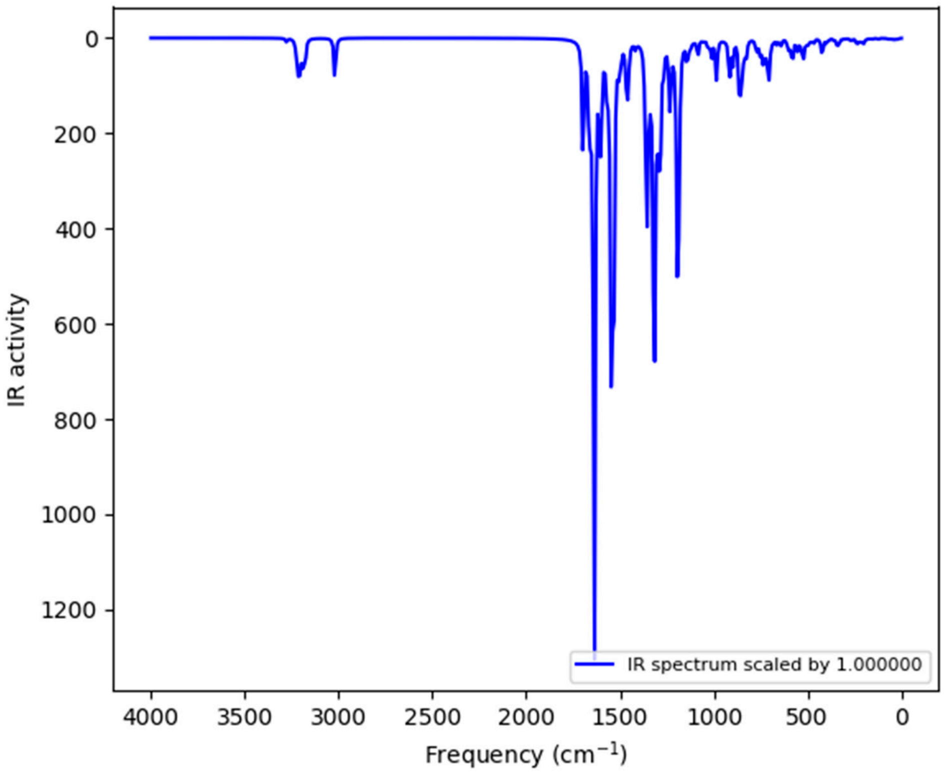

Figure S4. Theoretical IR spectra of di(FPh-ThAz-An)-TPA along with theoretical vibrational frequencies.

Frequency, cm^{-1}	Intensity
4.2271	0.0646
4.739	0.0083
5.0224	0.1351
11.4016	0.0332
13.4307	0.109
15.401	0.0919
17.1202	0.7089
20.2491	0.338
25.0976	0.4342
26.5376	1.7597
36.9618	1.1509
37.9342	0.7657
41.5241	0.6158
43.1503	0.9915
52.9263	1.7002
61.8369	0.5278
66.0212	0.4195
67.5683	0.9414
70.2814	0.9192
78.3448	0.2321
86.1931	0.1097
92.0043	0.2985
94.5567	0.0483
121.5862	2.1077
129.4555	0.2752
134.7887	0.2067
154.2212	1.9875
166.4678	0.9651
181.6251	1.5339
193.3431	1.3956
203.2433	13.2934
213.6729	4.2451
219.0436	2.1144
228.2833	5.8684
234.6538	5.5148
239.3343	6.6185
254.4363	0.4684
263.6721	2.8796
273.3383	3.4758
297.5608	1.2009
312.9813	1.3207

331.8154	5.7492
333.583	4.4936
340.8883	7.2175
342.2801	9.335
353.5755	0.2409
358.6678	2.7217
362.349	0.377
387.4435	2.5517
394.5149	1.4397
399.5193	7.5759
409.5767	0.236
414.9438	2.6375
418.6475	8.17
419.9396	0.0263
420.7648	8.4636
422.2867	0.1345
422.8158	1.7874
424.5864	16.8468
427.971	2.3609
433.0738	0.9248
467.5489	10.8507
476.8095	1.4116
485.451	5.9403
497.4512	7.901
503.327	6.8918
511.8238	4.582
518.2746	4.2982
524.0498	47.5578
533.4582	9.2923
539.8283	2.0967
550.9131	14.8289
556.2919	15.3086
578.268	34.7029
586.82	25.4088
595.1104	4.8631
599.0084	4.8051
601.4874	12.4481
608.7335	5.9787
631.3151	0.3515
640.517	6.7086
642.9655	5.6726
643.9733	1.3329

649.5854	0.7118
650.5386	2.5181
655.762	0.3042
664.2276	9.3641
668.3644	1.6573
684.1016	3.6384
689.2551	1.8271
699.1232	10.9494
706.4436	62.1567
707.6369	7.6389
711.4275	25.2079
713.382	8.308
721.4134	8.6761
726.6924	25.9404
736.5065	30.4089
740.2512	16.8146
748.2623	10.2489
753.8993	22.671
767.6049	17.8265
774.9408	14.6972
820.2192	1.0097
824.4319	5.9494
825.5781	4.2105
827.0669	16.2951
828.7658	8.4896
835.7262	11.9087
839.1651	10.6203
843.2506	12.2877
844.5211	1.2775
845.619	1.4923
849.3698	0.3463
851.5364	35.9935
854.2835	22.9642
854.6438	33.0228
857.5802	0.6439
859.0531	18.08
861.2978	14.7541
862.9139	26.8566
865.0088	64.0782
899.1862	45.7729
899.8146	8.1392
915.4859	2.2923

916.4017	65.3085
917.6007	29.2625
940.9641	0.8849
943.1981	0.1943
944.6323	1.2961
945.1945	0.0162
950.9769	0.5499
960.1292	0.1501
962.9545	0.5188
964.8478	0.4447
973.5282	0.8041
977.3035	0.7312
977.7386	0.2616
980.2039	0.3834
984.3483	42.8625
987.0608	39.2395
989.8009	0.9095
990.8308	8.3544
991.3532	6.0101
996.004	0.0343
1011.4671	15.1745
1012.6257	26.2512
1016.3001	0.5221
1028.794	0.0304
1029.4544	5.8663
1029.5557	4.1183
1030.5901	6.6763
1031.4487	0.323
1031.9347	2.4912
1054.5531	3.9553
1084.8286	23.0112
1085.5118	17.1671
1110.297	4.3043
1125.129	11.4122
1128.1843	7.41
1139.9173	8.9184
1141.5833	4.6797
1142.4985	29.2116
1145.0197	10.9488
1184.4569	97.261
1184.6411	84.2184
1187.1499	1.6907

1190.6516	60.8163
1191.9819	474.8897
1198.2327	16.5588
1200.1306	39.0369
1201.8941	10.2155
1216.7329	9.2937
1229.1266	33.5876
1231.813	12.6751
1233.872	81.284
1237.5614	38.0907
1266.429	7.9708
1268.7951	43.352
1281.6736	0.3801
1283.2932	37.4379
1284.67	118.5466
1288.8777	166.52
1301.4393	162.7848
1314.3509	43.7893
1316.4623	279.6281
1316.9769	238.1916
1320.1304	317.5314
1323.5442	12.6906
1326.7708	6.2302
1327.4399	4.6583
1330.5267	6.6994
1338.9921	34.2939
1343.2047	34.5613
1346.0933	26.0217
1347.1901	20.9312
1356.5401	371.5293
1362.8758	44.2219
1363.4544	7.3885
1364.4876	27.6065
1367.0307	25.5067
1417.1137	8.605
1417.8193	5.8932
1450.8549	11.0706
1453.4969	19.0389
1457.3877	6.0928
1459.127	10.8845
1462.7081	139.4332
1472.5975	8.1315

1495.9676	41.3624
1507.6716	39.3301
1509.9409	5.2878
1532.3129	353.0201
1535.2455	154.0501
1536.9371	243.7608
1546.8942	583.01
1550.5635	39.0499
1562.2602	8.5019
1565.8255	34.3677
1567.5544	25.5682
1572.3119	42.9418
1600.6343	212.4649
1604.2636	9.5399
1609.5662	18.7261
1611.2892	69.2059
1636.1453	1244.0275
1638.0356	44.9431
1639.0768	2.8862
1641.965	10.8569
1643.4309	119.0346
1652.5274	32.7629
1659.558	93.0719
1664.2675	66.1336
1667.2205	34.8992
1667.5989	18.307
1696.4079	266.1935
1697.2916	26.4722
3018.752	46.0733
3020.0343	53.2414
3177.1622	15.3812
3177.4377	13.7813
3179.2223	22.6477
3183.7508	3.7016
3188.2376	25.6784
3190.8355	7.0842
3191.5844	7.4748
3191.8982	5.5791
3195.7025	9.2388
3202.0218	7.0875
3204.0062	28.6086
3205.1229	4.2723

3206.9684	7.0878
3207.7648	5.0802
3210.2556	11.7854
3210.5771	12.8746
3213.3664	6.392
3214.4748	6.3203
3214.9079	18.6945
3215.5006	3.7737
3216.7001	6.6799
3219.6265	8.959
3221.1069	4.7178
3221.527	6.1441
3222.7073	5.1559
3223.5891	1.6248
3226.3975	2.6382
3231.3211	2.942
3231.5412	2.3527
3276.4565	2.4322
3278.0467	4.9463

3,5-di(diMeTPA)-1,2,4-ThdiAz:

Optimized geometry

C	8.718025000000	4.869810000000	3.039975000000
C	8.310176000000	3.682048000000	2.201716000000
C	7.069238000000	3.646608000000	1.551424000000
C	6.676971000000	2.546494000000	0.793231000000
H	6.402499000000	4.502087000000	1.625266000000
C	7.518657000000	1.431173000000	0.679541000000
H	5.718579000000	2.547678000000	0.284335000000
H	8.377798000000	4.760210000000	4.077443000000
H	8.286495000000	5.798656000000	2.654896000000
H	9.805340000000	4.987338000000	3.066218000000
C	8.761814000000	1.453280000000	1.325001000000
N	7.133232000000	0.300565000000	-0.103718000000
C	9.148409000000	2.567745000000	2.065376000000
H	10.118621000000	2.565677000000	2.555683000000
H	9.423366000000	0.597626000000	1.238367000000
C	8.075015000000	-0.222543000000	-1.042254000000
C	5.861086000000	-0.273067000000	0.035420000000
C	8.353558000000	-1.595490000000	-1.088875000000
C	8.750310000000	0.635420000000	-1.919879000000
C	5.242582000000	-0.934281000000	-1.046511000000

C	5.168555000000	-0.205298000000	1.267645000000
C	9.277302000000	-2.092677000000	-2.004640000000
H	7.840711000000	-2.268794000000	-0.409776000000
C	9.684399000000	0.126257000000	-2.818799000000
H	8.538562000000	1.699324000000	-1.894083000000
C	3.987768000000	-1.502730000000	-0.895365000000
H	5.750895000000	-0.988588000000	-2.001852000000
C	3.915039000000	-0.770594000000	1.405269000000
H	5.635583000000	0.286138000000	2.113168000000
C	9.966099000000	-1.244674000000	-2.882552000000
H	9.475837000000	-3.161243000000	-2.027352000000
H	10.195258000000	0.808528000000	-3.493587000000
C	3.293317000000	-1.433817000000	0.326713000000
H	3.523800000000	-2.000805000000	-1.743014000000
H	3.392851000000	-0.726826000000	2.355094000000
C	10.996767000000	-1.786888000000	-3.843633000000
C	1.983276000000	-2.038125000000	0.445151000000
H	10.746386000000	-2.800767000000	-4.169809000000
H	11.989819000000	-1.831770000000	-3.378879000000
H	11.083956000000	-1.157070000000	-4.733916000000
N	1.296382000000	-1.998175000000	1.544424000000
H	1.586035000000	-2.541000000000	-0.443961000000
C	0.060083000000	-2.602502000000	1.551762000000
S	-0.876596000000	-2.622398000000	3.030942000000
N	-0.570812000000	-3.207116000000	0.569150000000
N	-2.141071000000	-3.449493000000	2.289355000000
C	-1.785603000000	-3.653745000000	1.029756000000
N	-2.563155000000	-4.441271000000	0.187615000000
C	-3.684117000000	-4.095157000000	-0.322724000000
C	-4.436064000000	-2.832113000000	-0.315233000000
H	-4.182252000000	-4.884924000000	-0.895960000000
C	-3.953791000000	-1.595878000000	0.157536000000
C	-5.732614000000	-2.855429000000	-0.860817000000
C	-4.733857000000	-0.452259000000	0.098418000000
H	-2.961677000000	-1.524195000000	0.583459000000
C	-6.524222000000	-1.718645000000	-0.922804000000
H	-6.123065000000	-3.790985000000	-1.253912000000
C	-6.037945000000	-0.489140000000	-0.438591000000
H	-4.341318000000	0.483314000000	0.479570000000
H	-7.516808000000	-1.770867000000	-1.354914000000
N	-6.829913000000	0.672095000000	-0.491045000000
C	-6.232541000000	1.957765000000	-0.651676000000

C	-8.251017000000	0.588790000000	-0.390517000000
C	-6.635731000000	3.032248000000	0.153023000000
C	-5.245482000000	2.175466000000	-1.623154000000
C	-9.068263000000	1.290841000000	-1.286629000000
C	-8.855353000000	-0.182454000000	0.612488000000
C	-6.065727000000	4.291059000000	-0.018328000000
H	-7.394188000000	2.874189000000	0.912624000000
C	-4.671046000000	3.435555000000	-1.770634000000
H	-4.927090000000	1.353447000000	-2.255844000000
C	-10.454843000000	1.223467000000	-1.173764000000
H	-8.611643000000	1.886501000000	-2.070229000000
C	-10.243025000000	-0.254986000000	0.701823000000
H	-8.233894000000	-0.726837000000	1.315952000000
C	-5.071990000000	4.520007000000	-0.979219000000
H	-6.390778000000	5.109340000000	0.619474000000
H	-3.903438000000	3.580366000000	-2.526772000000
C	-11.071499000000	0.449791000000	-0.181966000000
H	-11.069969000000	1.773837000000	-1.881397000000
H	-10.690345000000	-0.862322000000	1.484764000000
C	-4.474378000000	5.893102000000	-1.173391000000
C	-12.575041000000	0.399203000000	-0.051380000000
H	-4.470702000000	6.463448000000	-0.239601000000
H	-5.044572000000	6.476623000000	-1.907421000000
H	-3.444152000000	5.833852000000	-1.536902000000
H	-13.066214000000	0.573736000000	-1.013318000000
H	-12.941188000000	1.165212000000	0.643960000000
H	-12.911979000000	-0.569799000000	0.329103000000}

2-diMeTPA-1,3,4-ThdiAz:

Optimized geometry

C	-5.765354000000	-0.136916000000	0.076972000000
N	-6.314929000000	-1.318430000000	-0.084323000000
N	-7.678594000000	-1.284485000000	-0.087152000000
C	-8.155479000000	-0.083884000000	0.084400000000
S	-6.951110000000	1.157640000000	0.274006000000
H	-9.216780000000	0.128945000000	0.106783000000
N	-4.426592000000	0.168820000000	0.143931000000
C	-3.578599000000	-0.699511000000	-0.309750000000
C	-2.146258000000	-0.502478000000	-0.228457000000
H	-3.923607000000	-1.628508000000	-0.777772000000
C	-1.276748000000	-1.473068000000	-0.759272000000
C	-1.576873000000	0.639011000000	0.373029000000

C	0.099320000000	-1.319189000000	-0.701969000000
H	-1.693473000000	-2.356893000000	-1.235759000000
C	-0.206084000000	0.801254000000	0.439252000000
H	-2.239659000000	1.385231000000	0.798145000000
C	0.664766000000	-0.175922000000	-0.098942000000
H	0.747465000000	-2.074717000000	-1.129754000000
H	0.211699000000	1.678772000000	0.918723000000
N	2.055305000000	-0.011613000000	-0.029871000000
C	2.640763000000	1.291330000000	-0.004970000000
C	2.927254000000	-1.143316000000	0.003050000000
C	2.253052000000	2.270320000000	-0.930350000000
C	3.636511000000	1.600836000000	0.930462000000
C	2.702698000000	-2.195061000000	0.901944000000
C	4.034345000000	-1.208552000000	-0.852677000000
C	2.839846000000	3.532848000000	-0.902568000000
H	1.494596000000	2.036748000000	-1.670421000000
C	4.227088000000	2.862131000000	0.935165000000
H	3.948220000000	0.847889000000	1.646865000000
C	3.562361000000	-3.290247000000	0.928246000000
H	1.851501000000	-2.152305000000	1.573659000000
C	4.895511000000	-2.302091000000	-0.803455000000
H	4.214405000000	-0.400943000000	-1.554624000000
C	3.837411000000	3.855610000000	0.027346000000
H	2.526542000000	4.277138000000	-1.630354000000
H	5.000415000000	3.080737000000	1.667205000000
C	4.678169000000	-3.364672000000	0.083421000000
H	3.368115000000	-4.097495000000	1.629936000000
H	5.747135000000	-2.335197000000	-1.478295000000
C	4.452750000000	5.233927000000	0.064526000000
C	5.628736000000	-4.536101000000	0.147334000000
H	3.881289000000	5.907263000000	0.715799000000
H	4.475699000000	5.688999000000	-0.930247000000
H	5.477203000000	5.205198000000	0.447170000000
H	5.110342000000	-5.458100000000	0.426962000000
H	6.417176000000	-4.368754000000	0.891981000000
H	6.122034000000	-4.703851000000	-0.814619000000}

di-BenzThAz-terTh:

Optimized geometry

C	-6.424813000000	-0.187046000000	0.023164000000
C	6.632648000000	1.003711000000	-0.022769000000
N	7.139510000000	-0.189198000000	0.005267000000

C	8.510792000000	-0.315967000000	-0.012731000000
S	9.143199000000	-1.980701000000	0.028438000000
N	9.407707000000	0.628368000000	-0.056348000000
C	10.761569000000	-1.316776000000	-0.018156000000
C	10.682065000000	0.101863000000	-0.060649000000
H	7.264310000000	1.897517000000	-0.059594000000
C	5.217344000000	1.216296000000	-0.007682000000
S	4.083805000000	-0.117706000000	0.045454000000
C	2.721250000000	0.985459000000	0.038559000000
C	4.553007000000	2.431973000000	-0.032614000000
C	3.151431000000	2.305067000000	-0.006501000000
H	5.076279000000	3.381038000000	-0.066286000000
C	11.992884000000	-1.975831000000	-0.017771000000
C	11.865637000000	0.857051000000	-0.103128000000
C	13.151945000000	-1.206057000000	-0.060314000000
H	12.047452000000	-3.059176000000	0.014747000000
C	13.087484000000	0.198295000000	-0.102601000000
H	11.801388000000	1.939516000000	-0.135513000000
H	14.119244000000	-1.699125000000	-0.060794000000
H	14.007682000000	0.773603000000	-0.135272000000
C	1.368357000000	0.490492000000	0.075672000000
H	2.471134000000	3.148906000000	-0.014510000000
S	0.000632000000	1.586643000000	-0.033106000000
C	0.925414000000	-0.815517000000	0.190813000000
C	-1.148372000000	0.264890000000	0.088762000000
C	-0.479009000000	-0.941476000000	0.198299000000
H	1.600395000000	-1.659398000000	0.275644000000
C	-2.567126000000	0.514261000000	0.065361000000
H	-0.991409000000	-1.892340000000	0.290361000000
S	-3.716334000000	-0.805496000000	0.060317000000
C	-3.231968000000	1.734539000000	0.042550000000
C	-5.072916000000	0.303887000000	0.033603000000
C	-4.632801000000	1.617313000000	0.024535000000
H	-5.326302000000	2.448730000000	0.008660000000
H	-2.710305000000	2.684895000000	0.045662000000
N	-7.427210000000	0.627373000000	0.001859000000
H	-6.553441000000	-1.278209000000	0.036166000000
C	-8.718531000000	0.176359000000	-0.017908000000
S	-9.173886000000	-1.590482000000	-0.095242000000
N	-9.731688000000	0.989759000000	0.012949000000
C	-10.853720000000	-1.097535000000	-0.073560000000
C	-10.933753000000	0.317690000000	-0.013982000000

C	-12.001886000000	-1.889874000000	-0.108862000000
C	-12.193618000000	0.937261000000	0.011646000000
C	-13.240982000000	-1.253136000000	-0.082632000000
H	-11.935199000000	-2.971898000000	-0.155046000000
C	-13.335580000000	0.147945000000	-0.022728000000
H	-12.247556000000	2.019662000000	0.057911000000
H	-14.146562000000	-1.851609000000	-0.108953000000
H	-14.314514000000	0.616837000000	-0.003294000000}

di(FPh-ThAz-An)-TPA:

Optimized geometry

C	11.394931000000	-0.267795000000	0.057002000000
C	12.593183000000	0.382142000000	0.238993000000
S	13.904688000000	-0.742324000000	0.274812000000
C	12.740412000000	-2.054831000000	0.043622000000
N	11.507617000000	-1.640502000000	-0.047980000000
C	13.144565000000	-3.464064000000	-0.041441000000
C	10.061452000000	0.355368000000	-0.021785000000
C	9.893242000000	1.751581000000	-0.051420000000
C	8.629863000000	2.318648000000	-0.131780000000
H	10.759854000000	2.405324000000	-0.030258000000
C	8.912030000000	-0.449176000000	-0.085014000000
C	7.643119000000	0.115001000000	-0.149665000000
H	9.031832000000	-1.526422000000	-0.090011000000
C	12.164548000000	-4.442484000000	-0.288827000000
C	14.479442000000	-3.870553000000	0.116914000000
H	12.778578000000	1.438357000000	0.363158000000
C	12.506025000000	-5.787208000000	-0.376217000000
H	11.135194000000	-4.126674000000	-0.411612000000
C	14.834298000000	-5.213591000000	0.030686000000
H	15.255289000000	-3.136241000000	0.312653000000
C	13.838892000000	-6.152599000000	-0.215075000000
H	11.760522000000	-6.551193000000	-0.567354000000
H	15.861430000000	-5.538817000000	0.151779000000
C	7.478314000000	1.511888000000	-0.155578000000
H	8.504818000000	3.395947000000	-0.167355000000
H	6.772779000000	-0.529133000000	-0.230996000000
N	6.234061000000	2.152495000000	-0.240940000000
C	5.208894000000	1.656234000000	0.351212000000
F	14.175556000000	-7.454872000000	-0.299425000000
C	3.874312000000	2.243079000000	0.242658000000
H	5.284363000000	0.762777000000	0.989172000000

C	2.798501000000	1.670166000000	0.939750000000
C	3.626069000000	3.379970000000	-0.548641000000
C	1.520135000000	2.208555000000	0.861168000000
H	2.971272000000	0.798444000000	1.566190000000
C	2.352765000000	3.917826000000	-0.641139000000
H	4.453167000000	3.819331000000	-1.095980000000
C	1.277481000000	3.339736000000	0.063198000000
H	0.705341000000	1.761964000000	1.420047000000
H	2.173435000000	4.787455000000	-1.263772000000
N	-0.019915000000	3.901639000000	-0.024981000000
C	-0.165747000000	5.322469000000	-0.065821000000
C	-1.169631000000	3.080078000000	-0.073851000000
C	-0.988646000000	5.921956000000	-1.030038000000
C	0.515105000000	6.129325000000	0.856923000000
C	-2.359316000000	3.478801000000	0.570285000000
C	-1.154944000000	1.859170000000	-0.771688000000
C	-1.133027000000	7.307733000000	-1.061060000000
H	-1.510070000000	5.298342000000	-1.748639000000
C	0.378543000000	7.515358000000	0.807195000000
H	1.147306000000	5.665249000000	1.606650000000
C	-3.491287000000	2.682914000000	0.516106000000
H	-2.379258000000	4.415060000000	1.116888000000
C	-2.292555000000	1.063627000000	-0.812573000000
H	-0.252493000000	1.545322000000	-1.283941000000
C	-0.448197000000	8.111221000000	-0.147267000000
H	-1.773389000000	7.760563000000	-1.812215000000
H	0.911111000000	8.129932000000	1.526934000000
C	-3.480645000000	1.456976000000	-0.175354000000
H	-4.404005000000	2.986039000000	1.017922000000
H	-2.266263000000	0.126089000000	-1.362626000000
H	-0.557559000000	9.190768000000	-0.178791000000
C	-4.661007000000	0.599798000000	-0.241538000000
N	-5.782420000000	0.918199000000	0.297687000000
H	-4.535317000000	-0.339675000000	-0.800864000000
C	-6.861581000000	0.027140000000	0.261539000000
C	-8.155323000000	0.564263000000	0.127509000000
C	-6.726660000000	-1.366469000000	0.407989000000
C	-9.268077000000	-0.260882000000	0.089794000000
H	-8.258643000000	1.640833000000	0.039463000000
C	-7.845368000000	-2.190107000000	0.387635000000
H	-5.744188000000	-1.796941000000	0.573990000000
C	-9.133290000000	-1.656721000000	0.218508000000

H	-10.261451000000	0.155636000000	-0.031097000000
H	-7.711073000000	-3.260794000000	0.515789000000
C	-10.331832000000	-2.499698000000	0.189343000000
N	-11.549363000000	-2.044643000000	0.071260000000
S	-10.255819000000	-4.266095000000	0.301908000000
C	-12.496697000000	-3.047891000000	0.060833000000
C	-11.979021000000	-4.315622000000	0.179730000000
C	-13.923049000000	-2.688312000000	-0.063389000000
H	-12.500517000000	-5.260242000000	0.216099000000
C	-14.921169000000	-3.662585000000	-0.233137000000
C	-14.310050000000	-1.338799000000	-0.011598000000
C	-16.262955000000	-3.308815000000	-0.339609000000
H	-14.653748000000	-4.712747000000	-0.293516000000
C	-15.648451000000	-0.969518000000	-0.118365000000
H	-13.543971000000	-0.582986000000	0.114184000000
C	-16.606596000000	-1.962648000000	-0.279320000000
H	-17.038428000000	-4.055020000000	-0.473022000000
H	-15.955162000000	0.069981000000	-0.077712000000
F	-17.906503000000	-1.613584000000	-0.383731000000

Table S1. Theoretical calculations of LUMO-HOMO levels of imines.

Code	HOMO [eV]	LUMO [eV]	LUMO- HOMO [eV]
**3,5-di(diMeTPA)-1,2,4-ThdiAz**	−5.145169328	−1.573646828	3.5715225
**di-BenzThAz-terTh:**	−5.54708466	−2.694764748	2.852319912
**di(FPh-ThAz-An)-TPA**	−5.231157984	−2.011481472	3.219676512
**2-diMeTPA-1,3,4-ThdiAz**	−5.306262	−2.041686348	3.264575652
